# Symmetry and Type of Peripheral Substituents Tune the Architecture of Gold-Supported Fluoro Phthalocyanine Films

**DOI:** 10.1021/acs.langmuir.6c02868

**Published:** 2026-09-21

**Authors:** Abdul Azeez, Jonathan Pinnock, Usha Kalra, Kai Hansen, Sanskruti Brahmbhatt, Hemantbhai H. Patel, Marius Pelmuş, Sergiu M. Gorun, Alexander Y. Fadeev

**Affiliations:** † Department of Chemistry and Biochemistry, 5971 Seton Hall University, South Orange, New Jersey 07079, United States; ‡ Center for Functional Materials, Seton Hall University, South Orange, New Jersey 07079, United States

## Abstract

A solution adsorption process for the facile production of films of C–H bonds free, fluorinated metal–organic phthalocyanines on gold is described. The effects of symmetry, electronic structures, and steric bulkiness, systematically varied within a series of electron-deficient perfluorinated zinc phthalocyanines, F*_n_* PcZn (*n* = 16, 40, 52, 64), have been modeled to account for experimental observations. The models reveal the underlying forces that contribute to Pc adsorptions and the structure of the resulting solid-supported uniform, lyophobic monolayer surfaces. The most energetic adsorption and the highest surface coverage, as revealed by X-ray photoelectron spectroscopy (XPS), adsorption isotherms, and contact angle measurements, occurred for *n* = 40 and 52 molecules that were arranged “edge-on-head-to-tail”, whereas molecules with *n* = 16 and 64 favored a “face-down” surface orientation. A structural model of surface coverage is proposed based on variation in interfacial interactions, ascribed to symmetry-imposed variations in molecular dipole moments and steric interactions as main factors that dictate the structure and properties of the constructed monolayers. The reported procedures and modeling might be used to reveal the factors that dictate the structure and properties of other solid-supported monolayers.

## Graphical abstract

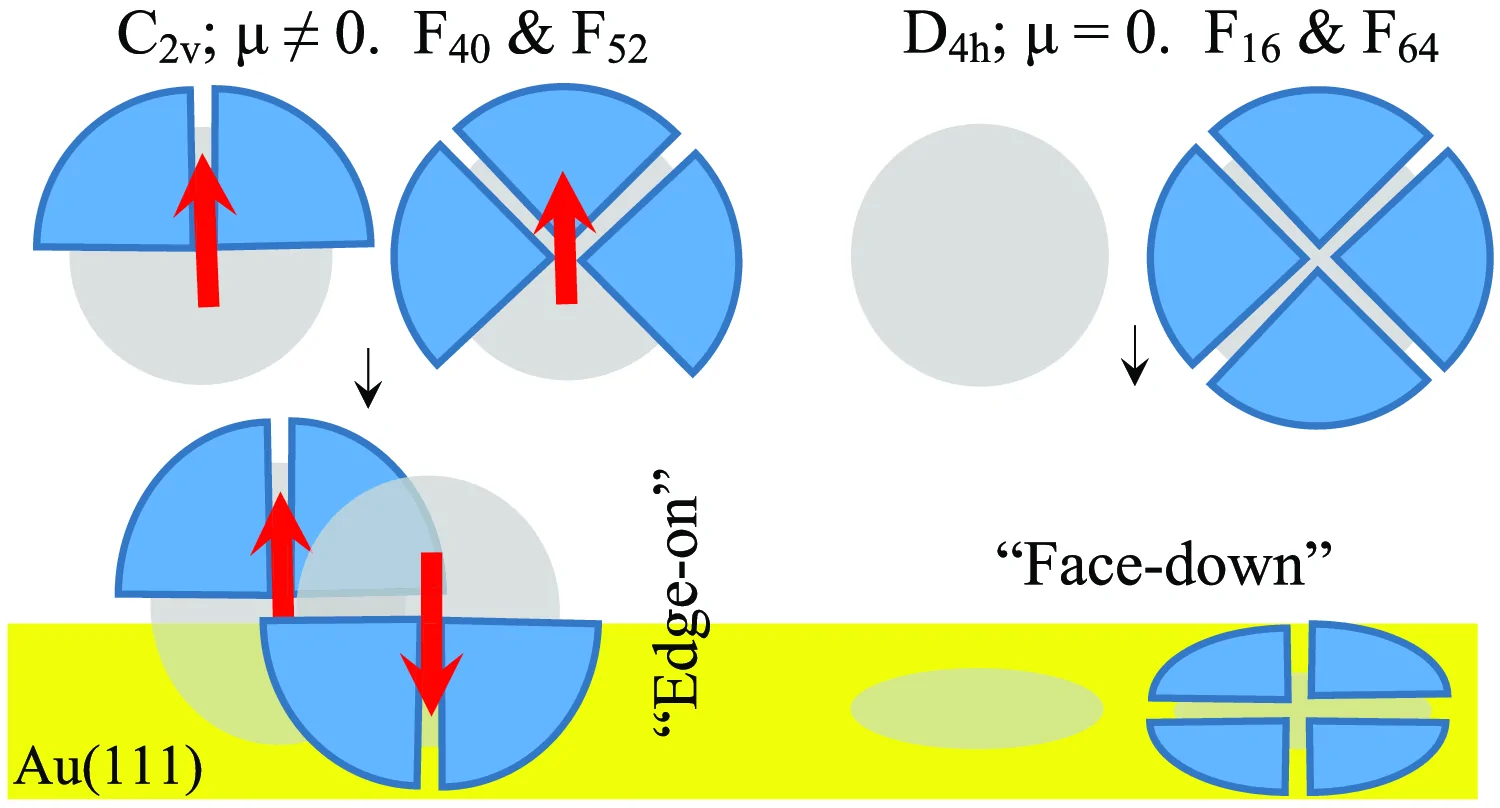

## Introduction

Phthalocyanines (Pcs) are highly conjugated macrocycles of interest in several technical areas, including catalysis and photocatalysis, dyes and pigments, information storage, conductive materials, etc. Pcs thin films, as recently reported, are of particular interest for sensors, energy conversion, information storage, nonlinear optics, and transistors.^1–9^ From a compositional point of view, Pcs bearing aromatic fluorine substituents have been considered advantageous based on (i) F-induced shift of their films toward n-type semiconductivity, candidates for organic field-effect transistors (OFETs), (ii) stabilization of film morphology, and (iii) enhanced Lewis acidity of coordinated metals that, in turn, enhances Pcs sensitivity toward electron-donor analytes such as ammonia.^10–15^ We have developed further a previously introduced class of Pcs exhibiting a mixture of aryl and iso alkyl fluorine substituents, resulting in extreme Pc electron deficiency,^16^ Figure 1, to complete the series of molecules containing 1, 2, 3, and 4 Pc quadrants containing the same fluoro alkyl substituents set. For the less hindered molecules, the formation of dimers in solution, relevant to the deposition procedure we report here, has recently been confirmed by electronic spectroscopy and magnetic circular dichroism studies, supported by DFT calculations.^17^

**Figure 1:**
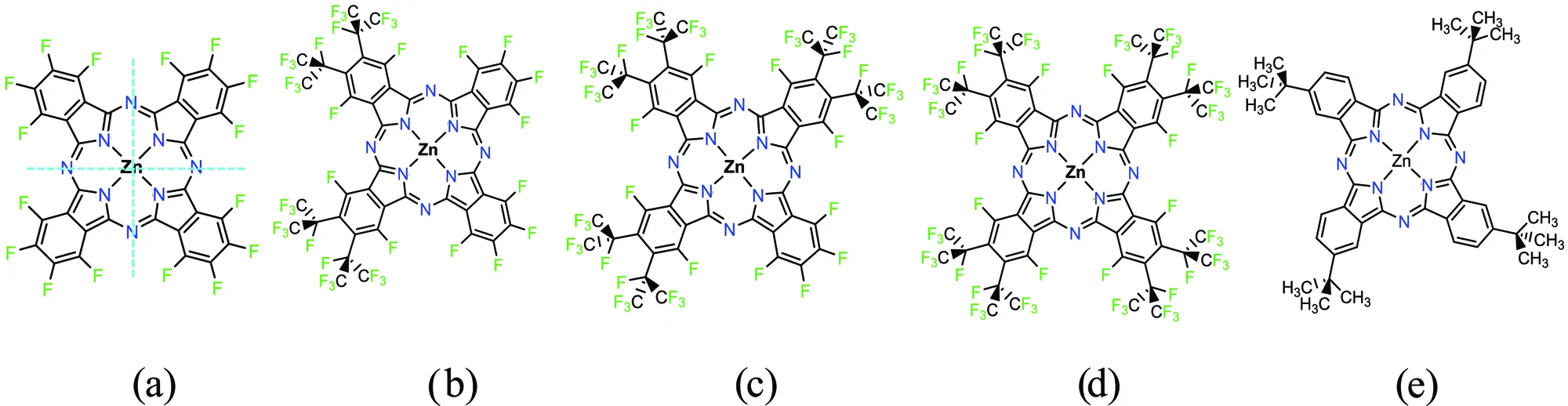
Phthalocyanine core structures. H_16_PcZn, not shown, was isostructural with F_16_PcZn. (a) F_16_PcZn, *z* = 0. The four quadrants of the Pc are marked by dashed lines; (b) F_40_PcZn, *z* = 2; (c) F_52_PcZn, *z* = 3; (d) F_64_PcZn, *z* = 4; (e) (*t*-Bu)_4_PcZn. The isomer shown is the metal complex of (*t*-Bu)_4_PcH_2_, characterized by XRD.^40^ The geometry of the Zn­(II) and 2H complexes of effective D_4h_ symmetry are likely to be similar, since the Pc structural changes, imparted by the replacement of the two central H^+^ by Zn^2+^, are minimal.

The fluorinated materials are chemically and thermally robust and exhibit interesting properties ranging from efficient singlet oxygen production to aerobic catalysis to bactericide activity to photochromism and single molecular magnetism.^18–29^ Due to the presence of a highly polar metal center surrounded by the nonpolar peripheral groups, these molecules exhibited amphiphilic properties and, somewhat counterintuitively, demonstrated reasonably good solubility (∼10^–4^ M) in water–ethanol mixtures containing up to 95–98% water by volume.^30^ The macrocyclic ring substituents largely determine the assembly of these Pcs in the solid state, including in crystals. Specifically, the bulky perfluoroisopropyl (*R*_f_) groups disfavor stacking interactions, favoring single-site catalysis, and thus preventing photoexcited state deactivation via intermolecular interactions. The bulkiness also limits π–π stacking interactions and thus has a major influence on the structure of both crystalline and thin film materials constructed using the [(*R*_f_)_*x*_F_*y*_Pc]^2–^ ligand series.

The formation of Pc thin films relies upon the Pcs deposition on solid supports. Indeed, the assembly of macrocycles on well-defined surfaces has attracted significant attention due to their rich potential to create surface patterns with nanometer dimensions with specific electronic, sensor, magnetic, optical, and catalytic functionality. Recent reviews addressed the surface chemistry of Pcs and related compounds, focusing on the self-assembly of the macrocycles at solid/vacuum interface,^31,32^ as well as on the thermodynamics and kinetics of their 2-D and 3-D thin film growth including on the influence of deposition parameters (vapor vs solution deposition) on the structure, morphology, and physical properties of the films.^33^ The nature of supports, not just that of the Pcs, influences the properties of F*_n_* PcM films: for example, on SiO_2_, F_16_PcCu forms polycrystalline structures.^34^ On Au(111) and Au(100), H*_m_* F*_n_* PcMetal complexes (*n* = 0, 4, 8, and 16; *n* + *m* = 16) adopt a flat-lying first layer due to the planarity of the Pc and the aromatic F–induced increase in the interface dipole, and thus interfacial charge transfer. Metal-specific core hole screening effects at organic/metal interfaces were observed for both H_16_PcZn­(II) (*n* = 0) and F_16_PcZn­(II) (*n* = 16) deposited on Au(100).^35^

To observe clean intermolecular interactions within a monolayer and determine their role in molecular orientation on metal surfaces, a closed-shell metal such as Zn­(II) must be used, while the Pc must be designed to modulate, up to even prevent stacking interactions and interactions between the surface and the coordinated metal or the macrocycle π electronic cloud. Additionally, the Pc ring must retain its planarity to avoid Pc-surface decreased distances due to macrocycle doming or twisting. A precise and mild deposition method was also desirable. In the work reported here, these conditions were met by the progressive introduction of R_f_ groups (*i*-C_3_F_7_) at the Pc ring periphery, Figure 1. The room temperature equilibrium solution adsorption method was used as the deposition technique. This deposition technique offered control in preparation of surfaces with monolayer and submonolayer coverage, and had a distinct advantage over frequently used vapor deposition,^31–33^ dip-, spin-, and spray-coating techniques,^33^ by allowing for deposition of Pcs on planar substrates as well as on substrates of complex topography, e.g., nanoparticles, fibers, and porous surfaces. The solution adsorption preparation of (*R*_f_)_*x*_F_*y*_PcZn (*x*, *y* = (0, 16), F_16_PcZn, and (8, 8), F_64_PcZn) supported on mica sheets and polymer films,^30^ textile fibers,^30^ mesoporous silicas,^30^ titanium wafers^36^ and titania nanoparticles.^37^ was reported recently.

The effects of point-group symmetry variability, electronic structure, and steric bulkiness of aromatic and aliphatic perfluoro substituents grafted on Pcs and thus present in their supported films are unknown, making it worth investigating, especially as we consider the expected chemical and thermal robustness of such films. The range of Pcs bearing F substituents, Figure 1, represented by the (R_f_)_*x*_F_*y*_PcZn can be classified based on the structural parameter *z*, the number of quadrants occupied by *R*_f_ groups: H_16_PcZn (*z* = 0), F_16_PcZn (*z* = 0), F_40_PcZn (*z* = 2), F_52_PcZn (*z* = 3). and F_64_PcZn (*z* = 4). An electron-rich Pc, comprising four *t*-Bu (*t*-C_4_H_9_) substituents, was also included in our study, Figure 1e. The electronic structure of the Pc-rings varies from electron-rich, imparted by the *t*-C_4_H_9_ groups, to progressively higher electron-deficient as *z* increases. It should be noted that, due to the π back-bonding ability of aromatic F, alkyl *R*_f_ groups are, overall, more electron-withdrawing. Thus, as the value of *z* increases, the replacement of F by *R*_f_ results in a more electron-deficient Pc, enhancing its ability to bind to a metallic surface, but, on the other hand, the bulkiness of the *R*_f_ groups acts in the opposite direction.

The contributions of molecular symmetry, electronic structures, polarity, and steric hindrance have been modeled to account for the experimental observations and to reveal the underlying forces that contribute to the adsorption process, molecular interactions, and organization of adsorbed macrocycles on solid supports based on progressively more complex ring-shaped macrocyclic platforms.

## Materials and Methods

Solvents were obtained from commercial sources and used without further purification, unless stated otherwise. Gold-coated wafers (Sigma-Aldrich) were ∼100 nm thick layers of highly crystalline Au(111) supported on Si(100). Prior to the adsorption of Pcs, the gold wafers were cut into ∼1 × 1 cm^2^ pieces and soaked for at least 2 h in a freshly prepared Piranha solution. *Caution: Piranha solution, a 3:1 mixture of 98% sulfuric acid and 30% hydrogen peroxide, is a very strong oxidizer and should be handled with great care*. The wafers were then rinsed thoroughly with DI water, followed by ethanol, and dried in an oven at 60 °C. The resulting gold surfaces exhibited water contact angles below ∼5–10°, indicating the absence of organic contaminants.

### Zinc Phthalocyanines

Six zinc phthalocyanines were used in this study. Zinc phthalocyanine H_16_PcZn (97%) and zinc 2, 9, 16, 23 tetra-(*tert*-butyl) phthalocyanine (*t*-Bu)_4_PcZn (96%) were purchased from Sigma-Aldrich. The F*_n_* PcZn were synthesized by the tetramerization of phthalonitriles in the presence of zinc acetate using microwave irradiation, as described in our previous work.^16^ The crystal structures of F_16_PcZn,^38^ F_40_PcZn,^39^ F_52_PcZn,^21^ and F_64_PcZn^16^ have been reported. The molecular volumes of the Pcs (*V*_m, XRD_) were determined from their unit cells obtained via single-crystal X-ray diffraction (SCXRD). Void volumes were calculated with Mercury package^40^ using a 1.2 Å spherical probe and subtracting the void volumes from the unit cell volumes.^41^ The X-ray structure of (*t*-Bu)_4_PcZn has not been reported, but the structure of (*t*-Bu)_4_PcH_2_ was.^42^ For the estimation of the (*t*-Bu)_4_PcZn volume, the volume of the Zn coordination sphere, ZnN_4_, of H_16_PcZn^43^ was obtained by removing all remaining atoms, calculating the void volume, and subtracting it from the unit cell volume. The volume of ZnH_2_ of (*t*-Bu)_4_PcZn was calculated similarly, and the difference between the 2 volumes was applied as a correction factor to the molecular volume of (*t*-Bu)_4_PcH_2_. The calculated structure of (*t*-Bu)_4_PcZn was obtained by placing a Zn atom in the geometric center of the 4 coordinating Ns, after removing the 2 central Hs. The molecular volumes (*V*_m, vdw_) of the Pcs were also calculated from their structures optimized in ChemDraw, using the Marvin View software.^44^ The molecular cross-sectional areas (σ) were calculated for the “face-down” Pc orientation using van der Waals atomic radii. The monolayer thicknesses (*t*) in the “face-down” orientation were calculated as *t = V*_m_/σ separately using *V*_m, vdw_ and *V*_m, XRD_. The monolayer thickness in the “edge-on” orientation was calculated as a linear dimension of the Pc square: *t* = √σ. The dipole moments of the metal-free Pcs (μ) were calculated using the Marvin Sketch software.^44^ X-ray metrical parameters are reported with estimated standard deviations, esd, errors, usually at the third decimal place. The experimental, *V*_m, XRD_ cell volume errors are thus typically small, for example 0.03% (∼5 × 10^–4^ nm^3^) for F_64_PcZn, the relative error values being independent of the reported units.^16^ The *V*_m, vdw_ errors were computed by using the tabulated van der Waals atomic radii that are used to calculate interatomic contacts by Mercury.^40^ These values do not include solid state effects, dipolar interactions, H-bonding etc., which lead to “short-contacts”, a term that indicates distances between atoms that are shorter than the sum of their van der Waals radii. It is thus reasonable to consider that experimental errors in *V*_m, XRD_, which include the above effects and their associated extra errors, exceed the errors in *V*_m, vdw_. This meant that the 0.03% error in *V*_m, XRD_ could also be considered as the upper boundary for errors in *V*_m, vdw_. The molecular cross-sectional areas, thickness and dipole moments were also obtained from the molecular dimensions. The XRD errors in the bond-lengths of Pcs could be as high as 0.5% and this value was taken as the upper limit error in dipole moment and thickness calculations given their linear dependency on bond-lengths values.

### Solution Adsorption

The Pcs solutions of 1, 10, 20, 50, 100, and 200 μM concentrations in anhydrous ethanol were kept under an inert atmosphere. The highest concentration possible for H_16_PcZn was 40 μM. Freshly cleaned gold wafers were placed in contact with 5 mL of the Pc solution, equilibrated for 12 h at 25 °C, removed from the solution and then dried at 60 °C for 10 min. Adsorption measurements were performed in duplicate.

### X-ray Photoelectron Spectroscopy (XPS)

The XPS measurements were performed using a Thermo Scientific spectrometer and Al Kα microfocused radiation, spot size 400 μm, operating pressure 5 × 10^–9^ Pa, θ = 90° takeoff angle. The binding energy scale was referenced to Au 4f_7/2_ peak at 84.0 eV. The Avantage software and Gauss-Lorentz functions with Shirley background were used for spectra analysis and curve fits. The XPS measurements were repeated twice using duplicate adsorption trials. The errors in the atomic percentages for Au, N, F, and Zn were 2, 5, 7, and 20%, respectively. The errors in the binding energies were 0.05 eV (for C 1s, N 1s and F 1s) and 0.08 eV for Zn 2p, respectively. The attenuation of the Au 4f peak (84.0 eV) was used to quantify the amount of deposited phthalocyanines. The average thickness (*t*) of the films was calculated using the equation^45^

1 $$t=\ln\left(I_{0}/I\right)\lambda \sin\varphi$$

where *I*_0_ and *I* were the intensity of the Au 4f peak before and after the adsorption of Pcs, respectively. φ was the takeoff angle and λ - the mean free path of Au 4f photoelectrons in the adsorbed films. The value λ = 2 nm was calculated using the NIST electron inelastic mean free path database.^46^ The errors in thickness determined using eq 1 were 10% or better.

Samples of “bulk phthalocyanines” for XPS analysis were prepared by evaporating small drops (0.05 mL) of ∼300–500 μM Pcs solutions in ethanol deposited on gold wafers. Repeated 5–7 times the deposition-evaporation cycles produced colored films, visible with the naked eye, with a thickness of ∼0.1 μm, which was estimated from the total volume and the solution concentration used for deposition. For these samples, a very low intensity signal from the substrate (Au 4f) at ∼1–2 atomic % or less was noted.

### Contact Angles

Contact angles were obtained using a Kyowa DMo-502 goniometer and RO purified water and hexadecane as probe fluids. Dynamic advancing (θ_A_) and receding (θ_R_) angles were recorded while the probe fluid was added to, and withdrawn from the drop, respectively. The 5–7 measurements performed in different spots had a maximum error of 2°.

## Results and Discussion

The Pcs, Figure 1, have been selected to contain closed-shell Zn­(II). Its lack of ligand-field stabilization energy lowered the probability of tight axial bonding of solvents that might interfere with the adsorption of Pcs. The Zn center, however, exhibited variable Lewis acidity, which increased with the numerical value of the parameter *z*, Figure 1.

The Pc-rings’ effective symmetry was defined by considering the Pc ring and only the first atom of any substituent. For example, F_16_PcZn represented structurally the full effective symmetry, while in the case of F_40_PcZn, the Pc ring was substituted by 12 F and 4 C atoms. This structural abbreviation appeared reasonable since the aliphatic CF_3_ groups of the *i*-C_3_F_7_ substituents may occupy different positions due to the rotational disorder the *i*-C_3_F_7_, but their position had little relevance to the electronic structure of Pcs. Unlike aryl fluorines that exhibit through-space π-back-bonding and have σ withdrawing effects, aliphatic fluorines lack the former but retain the latter. The magnitude of the σ–effect, which is through-bond type, depends mainly on the number of F and their position in an aliphatic group, *R*_f_, (*n* chain vs iso structure, for example), but not on the *R*_f_ rotational position relative to the aromatic ring to which it is attached, a topic reviewed recently.^47^

Considering the effective symmetry, and, in the absence of ring doming or twisting, the F_16_PcZn, F_40_PcZn, F_52_PcZn, F_64_PcZn, and (*t*-Bu)_4_PcZn belonged to D_4h_, C_2v_, C_2v_, D_4h_, and C_4h_ point groups, respectively. The presence of a 4-fold axis resulted in centro-symmetry. The variation in the volume of Pc substituents, and in their positions, resulted in variations of molecular dimensions (cross-sectional area and thickness), dipole moments, and possible orientations in the adsorbed state on the gold surface. Table 1 summarizes up the structural parameters of the zinc Pcs under study.

**Table 1:** Molecular Parameters^a^ of the Pc Series

	Molecular volume (*V*_m_), nm^3^		Molecular cross-section (σ), nm^2^	Monolayer thickness (*t*), nm			
				“face-down”		“edge-on”	
Phthalocyanine	v-d-W	XRD	“face-down”	*t* = *V*_m,vdW_/σ	*t* = *V*_m,XRD_/σ	*t* = √σ	Dipole moment μ, D
(*t*-Bu)_4_PcZn	1.05	0.95	2.32	0.45	0.41	1.52	0.23
H_16_PcZn	0.67	0.58	1.72	0.39	0.34	1.31	0.0
F_16_PcZn	0.75	0.68	2.03	0.37	0.33	1.42	0.0
F_40_PcZn	0.98	0.98	2.47	0.40	0.40	1.57	5.14
F_52_PcZn	1.16	1.15	2.76	0.42	0.42	1.66	3.60
F_64_PcZn	1.42	1.60	2.94	0.48	0.54	1.71	0.0

a The details of the calculations and the precisions of the determination of the molecular parameters from the van der Waals models (v-d-W) and from the single-crystal X-ray diffraction data (XRD) are provided in the Materials and Methods section.

The differences between the XRD-determined and the van der Waals calculated molecular volumes were ∼10% or less, an error that did not affect the work conclusions. A plot of the van der Waals vs the XRD molecular volumes is shown in Figure S1, Supporting Information.

Insights into the composition of the Pcs thin films, as well as their surface chemistry, were provided by XPS. The XPS C 1s core level spectra of bulk/pure Pcs and their fits obtained via deconvolution considering symmetry-imposed C atoms equivalencies are overlaid in Figure 2. The full data of the C 1s peak fit analysis is given in Table S1.

**Figure 2:**
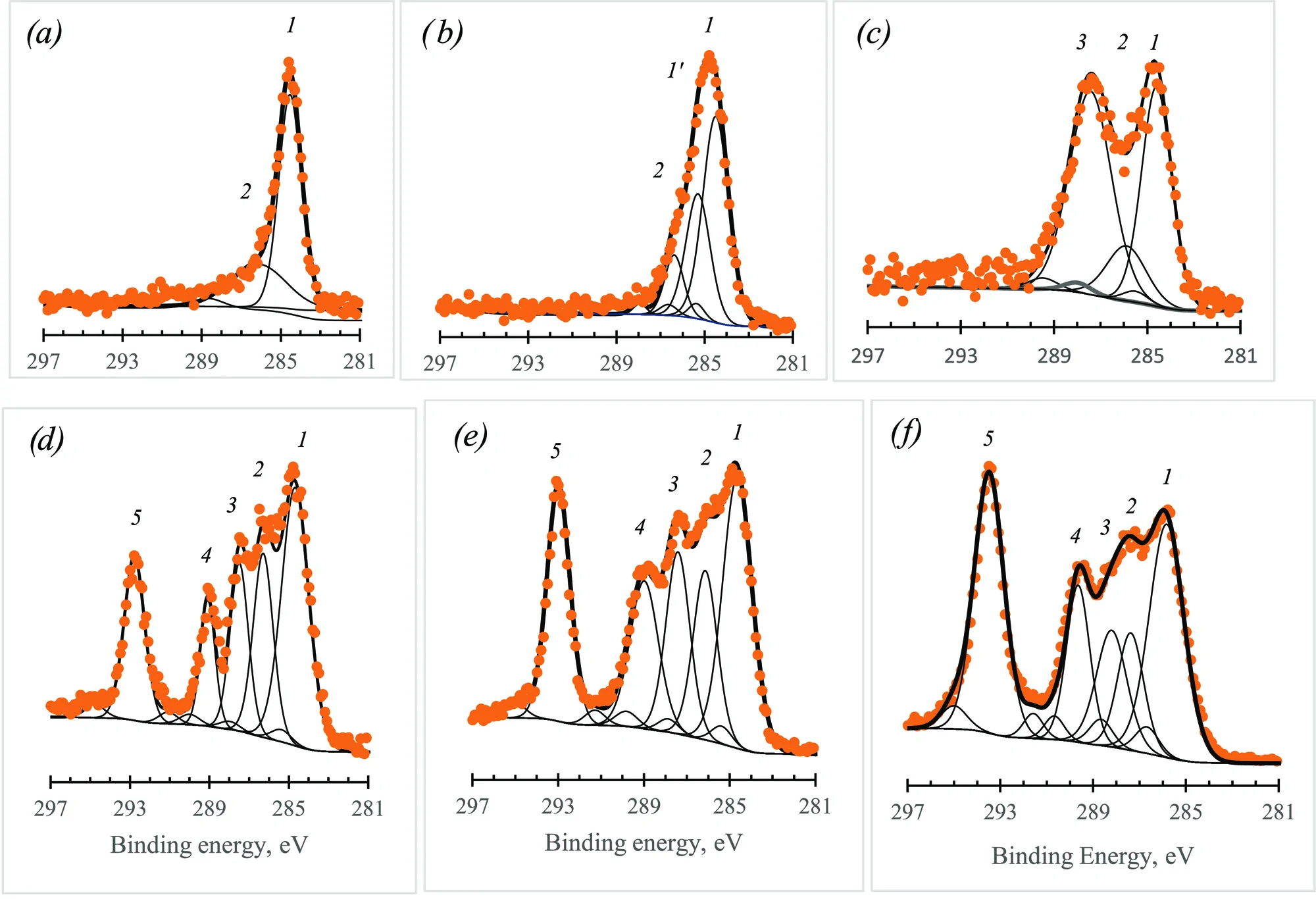
C 1s core level spectra of bulk Pcs and their numerical fits. (a) H_16_PcZn; (b) (*t*-Bu)_4_PcZn; (c) F_16_PcZn; (d) F_40_PcZn; (e) F_52_PcZn; (f) F_64_PcZn. Main peaks assignments: 1 – CC_sp2_, 1’ – CC_sp3_, 2 – CN, 3 – CF_sp2_, 4 – CF_sp3_, 5 – CF_3_. Satellites are not labeled for clarity. Table S1 summarizes the binding energies of all the peaks and the results of the peak fit analysis. *Y*-axes represent intensity in a.u.

The peak labels CC_sp2_, CC_sp3_, CN, CF_sp2_, CF_sp3_, and CF_3_ stand for C–C aromatic, C–C aliphatic, C–N, C–F aromatic, C–F aliphatic, and CF_3_ groups, respectively. In addition to the main peaks, the spectra exhibited weak satellites ∼1.7–1.8 eV above them in binding energy (Table S1). The inclusion of C 1s π–π* ″shakeup″ satellites, commonly found in conjugated aromatic molecules like Pcs, was necessary for accurate determination of the peaks positions, and peaks areas, and correct background subtraction. The data for F_64_PcZn, F_16_PcZn, H_16_PcZn, and (*t*-Bu)_4_PcZn, in close agreement with previous reports,^36,48,49^ provided a base for the C 1s peak assignment for hitherto unreported F_52_PcZn and F_40_PcZn, Table S1. The % areas of the individual peaks for each type of carbon obtained from the fit and their stoichiometric content were statistically indistinguishable, Table S1. The binding energies of Zn 2p_3/2_, F 1s, and N 1s peaks are plotted in Figure 3.

**Figure 3:**
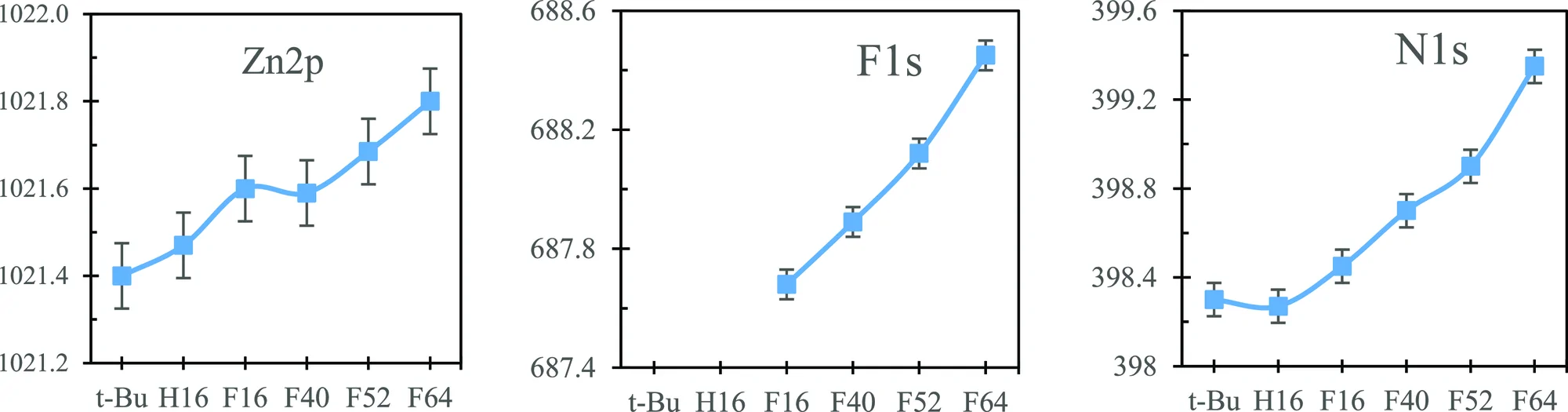
Binding energies (eV) of the core levels of Zn 2p_3/2_, F 1s, and N 1s peaks for pure Pcs. Symbols are connected to guide the eye. The Pcs are abbreviated based on their substituents.

The binding energy of Zn 2p_3/2_ increased gradually from 1021.4 eV for the electron-rich (*t*-Bu)_4_PcZn, reaching 1021.8 eV for the most electron-deficient F_64_PcZn, Figure 3. The 0.4 eV shift appeared to be large for a nitrogen-coordinated^50^ Zn­(II), underscoring the very high electron deficiency of the central metal. The Zn 2p spectra are shown in Figure S2. The N 1s spectra are shown in Figure S3. The azo and pyrrolic type Ns were not resolved; a single peak was observed.^36,49^ Similar to Zn 2p, its position was also shifted to higher binding energies as the number of electron-withdrawing R_f_ groups increased, Figure S4. The F 1s spectra (not shown) exhibited a single peak from the superposition of aromatic and aliphatic fluorines.^51^ Its binding energy gradually increased from 687.7 to 688.5 eV in the order F_16_PcZn-F_40_PcZn-F_52_PcZn-F_64_PcZn, reflecting the increase in its aliphatic F content. The plots in Figure 3 were only qualitative as the *x*-axis was arbitrary, viz. F_40_PcZn, F_52_PcZn, and F_64_PcZn were equally “spaced” by 12 F.

The C 1s, F 1s, N 1s, and Zn2 core level spectra of the Pcs adsorbed on Au provided insights into the electronic environment of fluorinated Pcs and their interactions with the substrate. As a representative example, Figure 4 presents an overlay of the C 1s regions of F_52_PcZn on Au as the amount of the adsorbed Pc increases.

**Figure 4:**
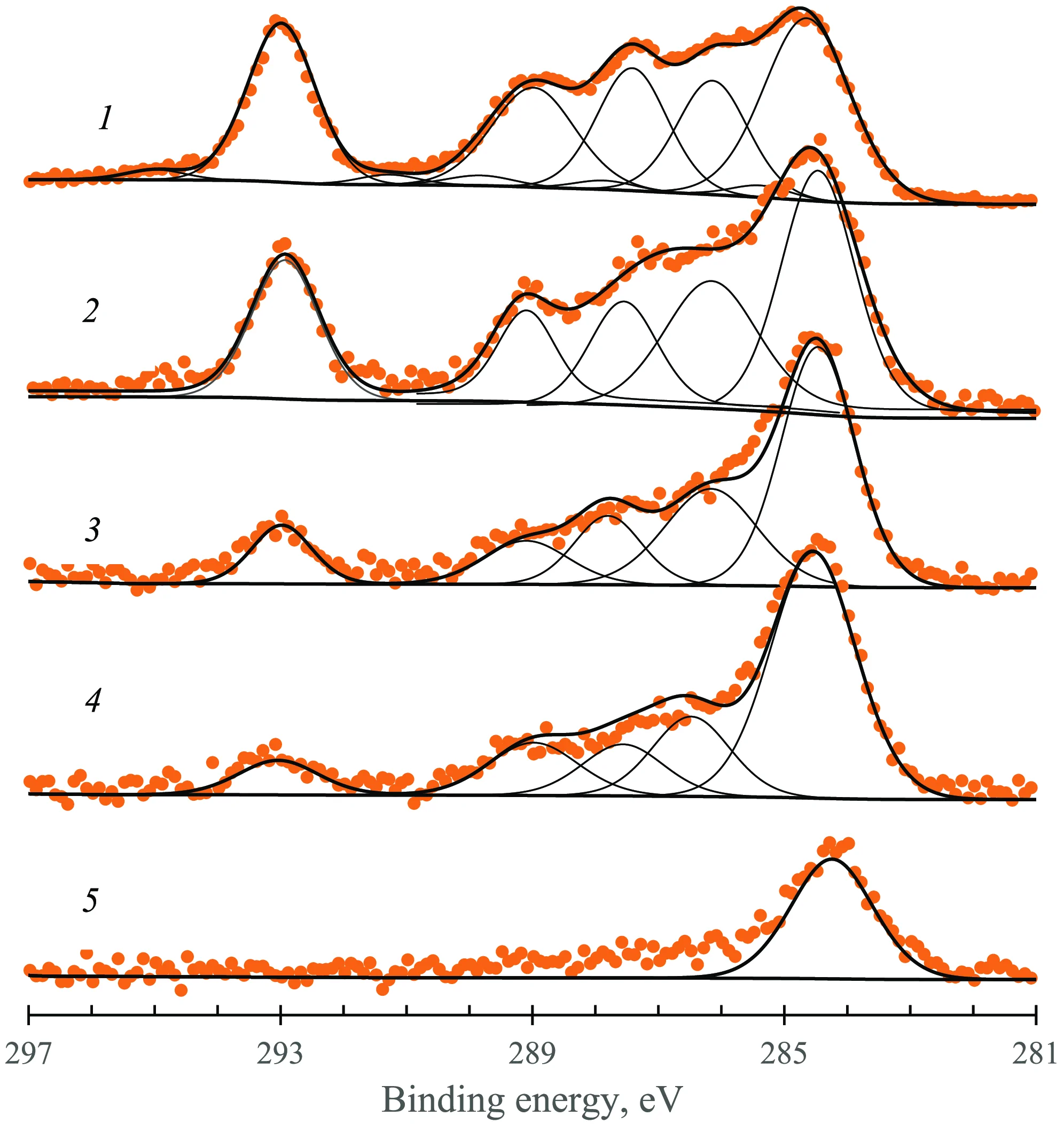
Core level C 1s spectra and their fits for F_52_PcZn supported on Au: (1) – bulk Pc, (2)–(4) samples obtained via adsorption from 200, 50, and 10 μM solutions of Pc in ethanol, (5) – bare Au. The spectra are offset for clarity. *Y*-axes represent intensity in a.u.

The peak intensities of the adsorbed and bulk Pc were in reasonable agreement, except for the peak at 284.8 eV (C–C), which, in the adsorbed films, was overrepresented due to contamination with adventitious carbon. Importantly, the similarity of the C 1s spectra of the gold supported Pcs at low surface coverages with that for the bulk indicated that no Pcs degradation/hydrolysis occurred upon the adsorption.

The changes in the Zn 2p region of the adsorbed Pcs were structurally dependent. For H_16_PcZn and F_16_PcZn, the Zn 2p binding energy shifted to lower values vs bulk Pcs, by 0.37 and 0.20 eV, respectively. Such significant shifts argued for the specific interactions between Zn and the gold surface, as opposed to π-π stacking, observed in bulk crystalline state. Notably, for F_40_PcZn, F_52_PcZn, F_64_PcZn, and (*t*-Bu)_4_PcZn, the position of the Zn 2p peak remained the same (within the error) as in the bulk Pcs, suggesting the absence of Zn–Au interactions. This was attributed to the difference in the molecular thickness of planar H_16_/F_16_PcZn and the Pcs containing bulky alkyl groups. Indeed, the van der Waals radii of Zn^2+^ and Au were^52^ 0.146 and 0.17 nm, respectively, and their sum was ∼0.31–0.32 nm, in agreement with the 0.33–0.38 nm thickness of monolayers of H_16_PcZn and F_16_PcZn in the “face-down” orientation (Table 1), thus allowing van der Waals contacts between Zn metal center and Au. Similar Zn 2p shifts were observed^35^ for H_16_PcZn and F_16_PcZn on a crystalline Au (100) face, but the Pcs have been deposited under the UHV conditions, not through solution adsorption equilibrium. The results of this work and work^35^ suggested the existence of a general Zn–Au wave functions overlap, independent of the Pc deposition mode, Au surface structure, and Pc electronic structures, as long as the “planar” (2D-type) Pc was used. In contrast, the other four Pcs, with *z* > 1, i.e., those containing multiple bulky *t*-C_4_H_9_ or *i*-C_3_F_7_ groups (3-D type), exhibited an increase in the Zn–Au distance, thus disrupting the direct Zn–Au wave functions overlap, rendering the Zn 2p core level binding energies for (*t*-Bu)_4_PcZn, F_40_PcZn, F_52_PcZn, and F_64_PcZn statistically indistinguishable from those of the bulk Pcs. Noteworthy, unlike the Zn binding energy, the F 1s and N 1s core level peak binding energies for both *z* = 0 (unencumbered, 2-D structures) and z > 1 (sterically encumbered 3-D structures) were statistically indistinguishable, underscoring the specificity of Zn–Au contact, and the absence of C, F, and N specific interactions with the metal surface.

Next, the XPS was used to reveal the chemical integrity of the adsorbed Pcs, as well as the concentration-dependence of N 1s, Zn 2p, and F 1s peaks, Figure 5.

**Figure 5:**
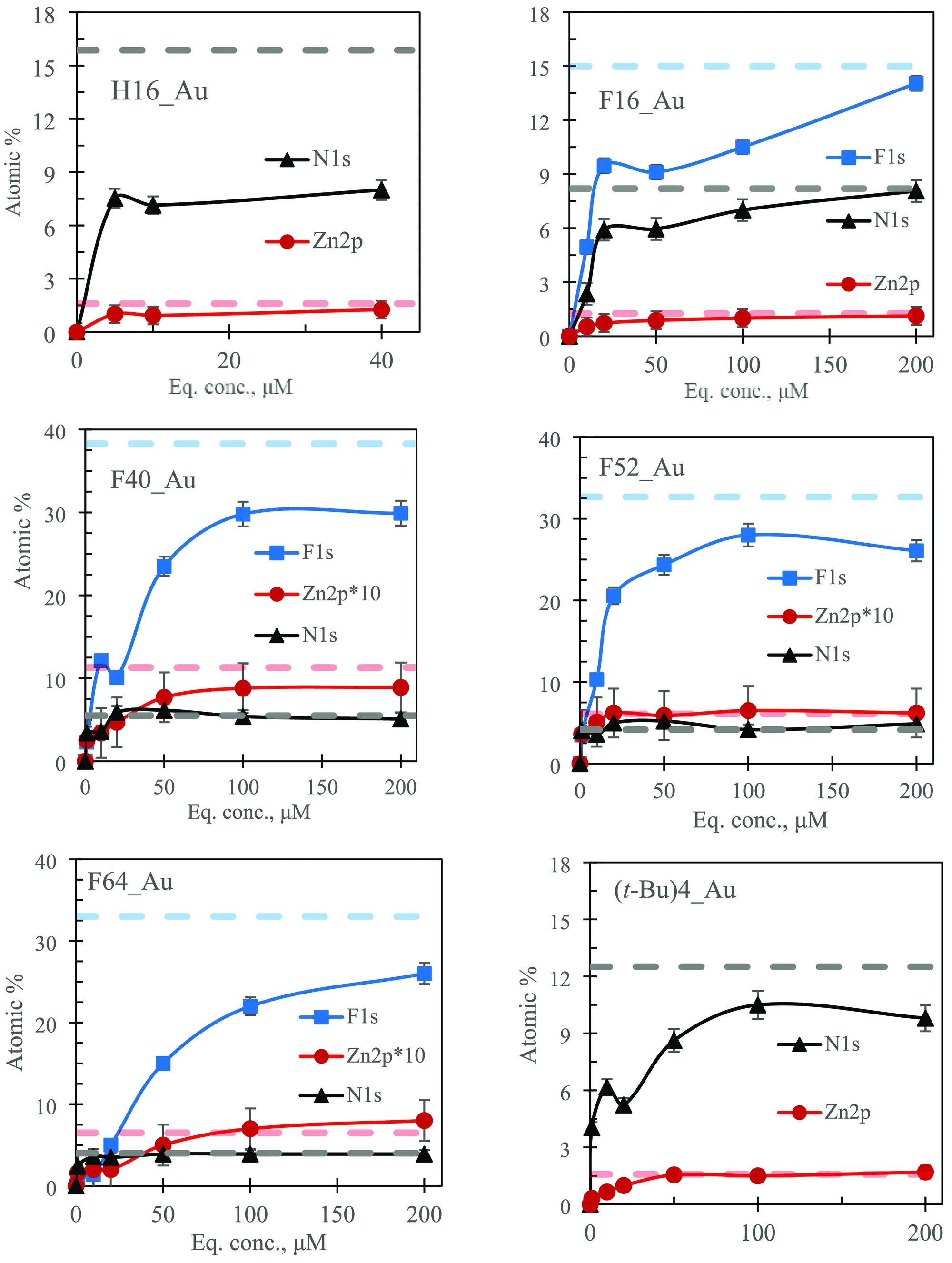
XPS-determined atomic % of N, Zn, and F of Pc films formed on Au surfaces vs variable equilibrium concentrations of Pcs in ethanol solutions. Symbols are connected to guide the eye. The dash lines correspond to the bulk Pcs. The Pcs rings substituents are used as abbreviations.

The surface compositions approached the atomic percentage values observed for bulk Pcs, while at saturation, the F/N/Zn atomic ratios matched them, and, within errors, the ratios predicted by the molecular formulas, Figure 6.

**Figure 6:**
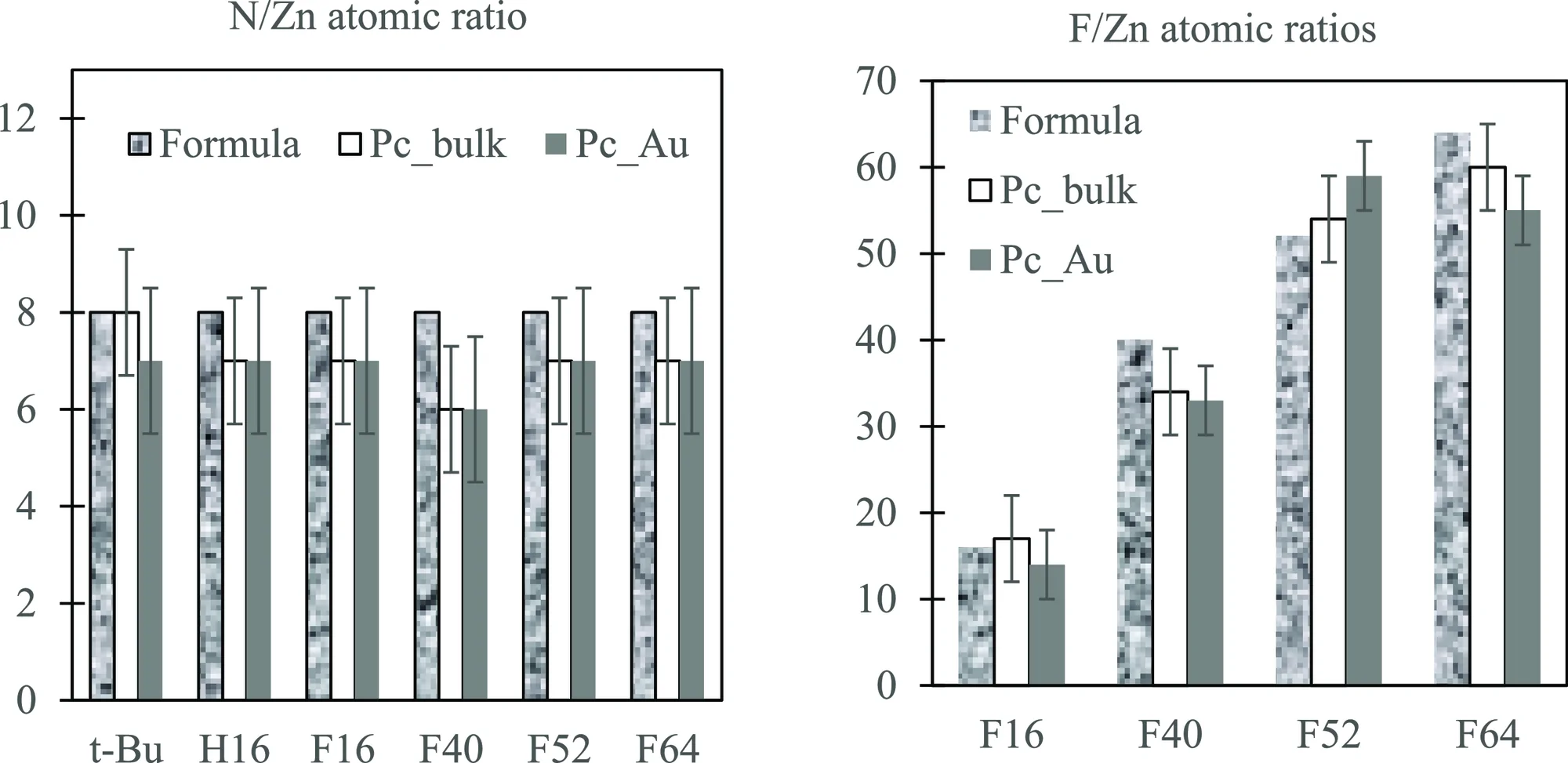
XPS atomic ratios obtained for bulk and gold-adsorbed Pcs in comparison with the formula ratios. *F_n_* labels are used as abbreviations.

Qualitatively, the data supported the notion that the adsorption process, irrespective of the equilibrium solution concentration, resulted in deposition of intact Pcs on the Au surface. Quantitative information was provided by measuring the concentration-dependence of the Pcs films’ thickness, which increased with the solution’s equilibrium concentration, Figure 7.

**Figure 7:**
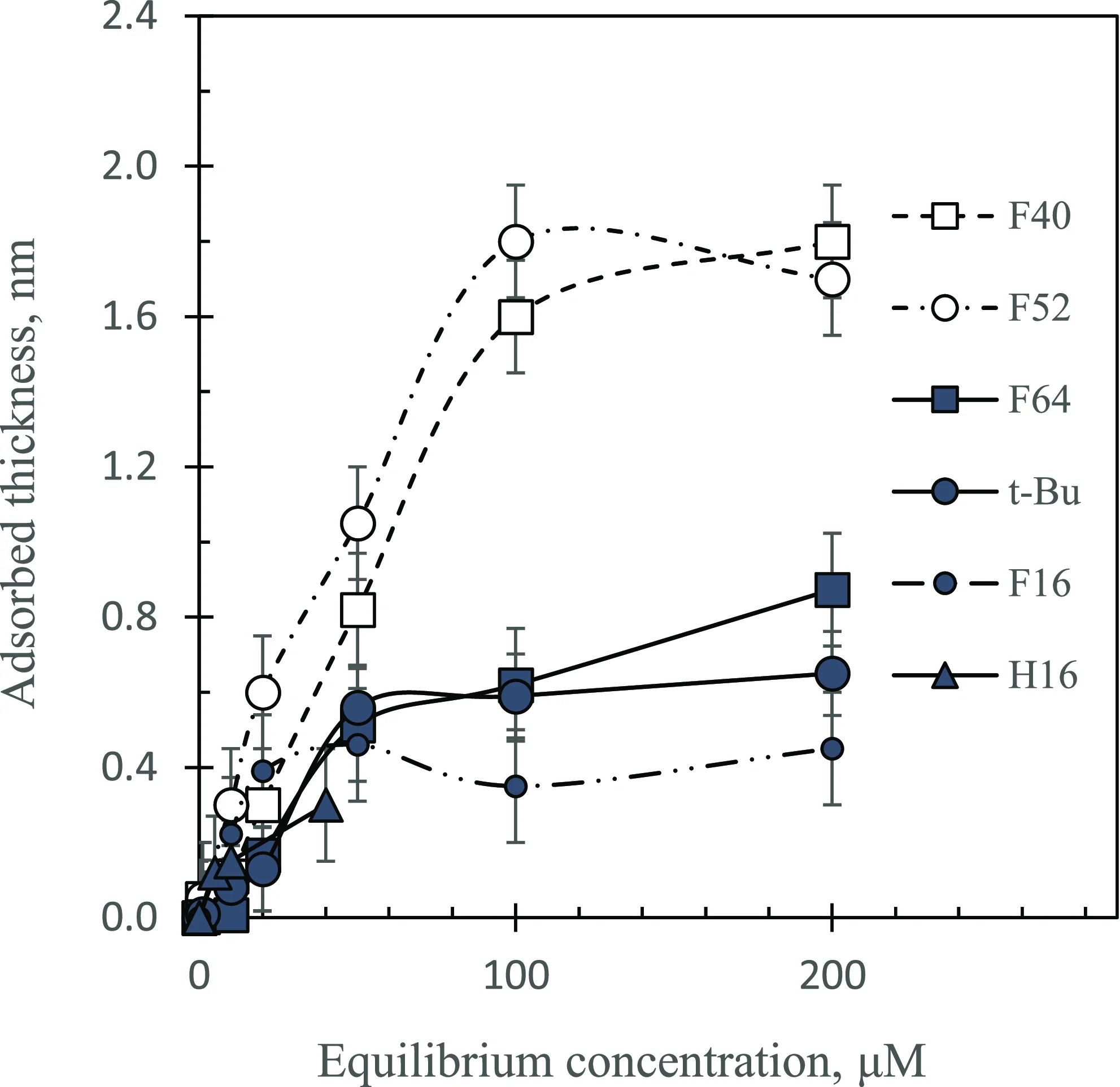
XPS-determined thickness of the adsorbed Pc films on Au vs Pcs solution concentrations. Symbols are connected to guide the eye. The Pcs rings substituents are used as abbreviations.

The thickness isotherms in Figure 7 showed regions of initial uptake followed by saturation plateaus after the solution concentration reached ∼50–100 μM. The plots in Figure 7 revealed a remarkable detail: the saturation adsorption of F_52_PcZn and F_40_PcZn yielded surfaces with thicknesses approximately twice as large as for the other Pcs. For the surfaces of H_16_PcZn, F_16_PcZn, (*t*-Bu)_4_PcZn, and F_64_PcZn, the thickness leveled off at ∼0.4–0.8 nm, in good agreement with the monolayer in a “face-down” orientation of the adsorbed molecules.

The adsorption of F_52_PcZn and F_40_PcZn yielded surfaces with thicknesses of ∼1.6–2.0 nm, consistent with an “edge-on” orientation of the Pcs on the surface. In principle, the larger values of thickness could also be associated with the formation of the “face-down” multilayers, as both complexes have benzene rings devoid of *R*_f_ groups and thus prone to stacking at 0.7–1.1 nm intermolecular distance, as evidenced by their single-crystal X-ray structures, Figure S6. However, the “edge-on” and the “face-down” orientations would result in different surface compositions and, hence, different wettability, probed by contact angle measurement, which was used to identify surface functionalities as well as the uniformity and lyophobicity of the Pc surfaces.

The contact angles using water and hexadecane probe fluids increased with the concentration of Pcs used for adsorption, indicating the formation of lyophobic surfaces, in agreement with the increase in the surface coverage revealed by XPS, Figures 5 and 7. Table 2 summarizes the maximal contact angles that were observed for gold surfaces exposed to 200 μM solutions of Pcs. Full contact angle data are provided in Figure S5.

**Table 2:** Highest Contact Angle Values for Pcs Supported on Au

	Contact angles^a^ (adv/rec), deg	
Surface	Water	Hexadecane
Bare Au	Spreads^b^	Spreads^b^
H_16_PcZn	90(1)/59(2)	Spreads^b^
F_16_PcZn	90(1)/66(2)	Spreads^b^
(*t*-Bu)_4_PcZn	92(20)/63(2)	12(2)/0
F_40_PcZn	98(1)/78(2)	37(1)/24(2)
F_52_PcZn	100(1)/80(1)	43(2)/27(2)
F_64_PcZn	95(1)/74(2)	24(2)/10(2)

a Standard errors are given in parentheses.

b The contact angles were below ∼5°.

The contact angles by hexadecane were of particular importance due to their unique sensitivity to the presence of CF_3_ functionalities,^53^ and thus to the values of *z*. The water contact angles followed the same general trend (Figure S5), yet the variation in hydrophobicity was more subtle than the variation in oleophobicity probed by hexadecane. The most oleophobic surfaces were produced, in order, by F_52_PcZn > F_40_PcZn ≫ F_64_PcZn, which appeared odd since F_64_PcZn had the highest number of oleophobic CF_3_ groups. Furthermore, the highest hexadecane contact angles, 43°/27° (adv/rec) observed for F_52_PcZn surfaces were noticeably smaller than the 56°/40° values expected for the surfaces of *i*-C_3_F_7_ groups,^54^ indicating the presence of less phobic surface functionalities. The hexadecane contact angles were analyzed using the Israelachvilli-Gee equation,^55^ treating the surfaces as mixtures of aromatic and aliphatic fluorines

$$\begin{array}{c} {\left(1+\cos\theta \right)}^{2}=f_{1}{\left(1+\cos\theta_{1}\right)}^{2}+f_{2}{\left(1+\cos\theta_{2}\right)}^{2}\\ f_{1}+f_{2}=1 \end{array}$$ 2

Using a 0° hexadecane contact angle value for aromatic CF (θ_1_) and 56° for *i*-C_3_F_7_ (θ_2_),^54^ the 43 ° advancing contact angle observed for F_52_PcZn corresponded to a surface composed (by area) of 36% aromatic F and 64% *i*-C_3_F_7_ (*R*_f_) groups, i.e., 36:64 surface mixture. For the surface of F_40_PcZn, the 37° advancing contact angle corresponds to a 49:51 F:*R*_f_ surface mixture. An inventory of the F and *R*_f_ groups exposed while the molecules can adopt any statistically equal orientations obtained by the formal 90 deg steps rotations of the Pcs planes around a perpendicular axis to the metal center (wheel rotation), thus exposing to the surface, in turn, each of the quadrants, yielded F:*R*_f_ = 50:50 for F_40_PcZn and 33:67 for F_52_PcZn. These numbers were in excellent agreement with the experimental values, supporting the “edge-on-head-to-tail” molecular arrangement shown in Figure 8.

**Figure 8:**
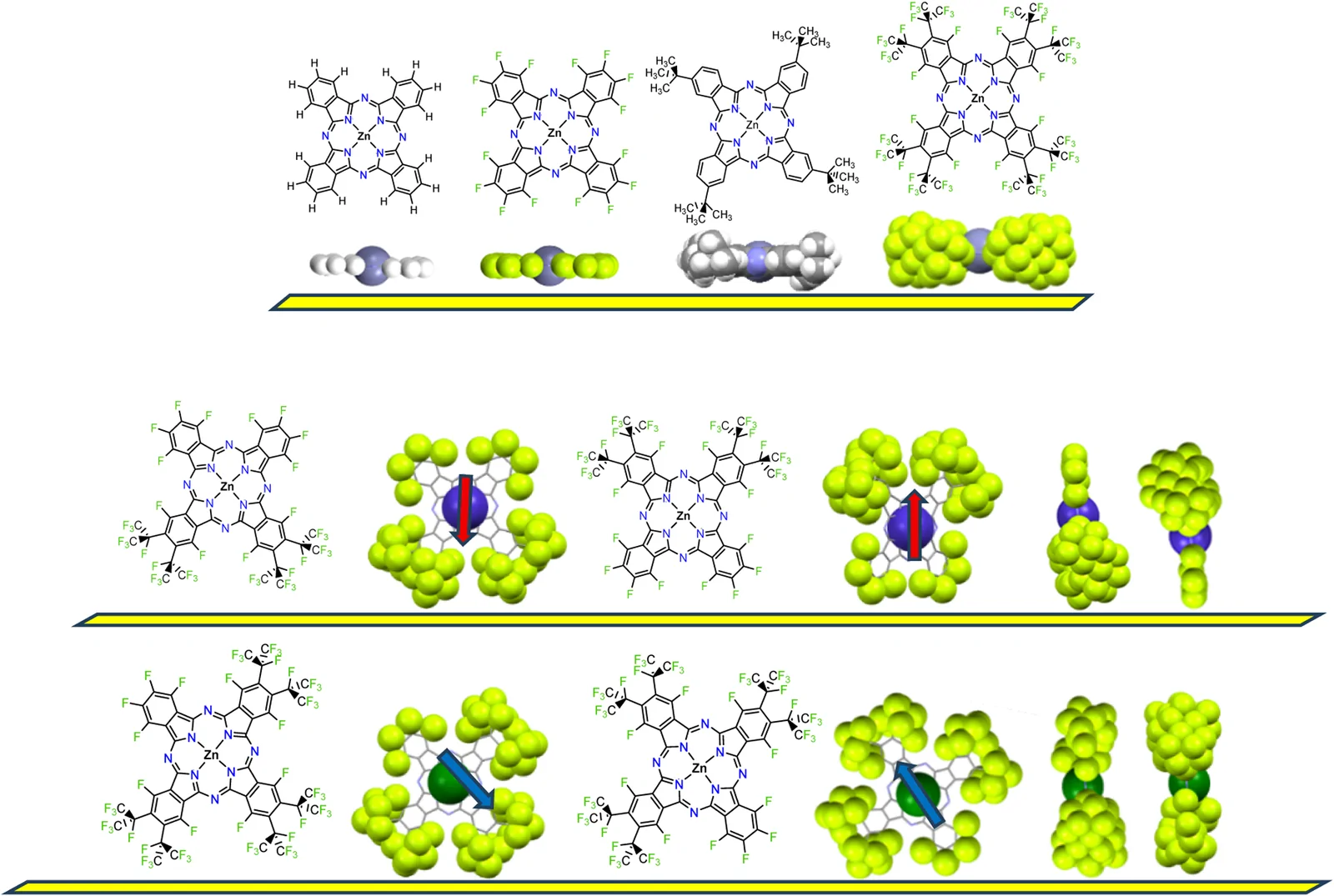
Proposed models of F_n_PcZn, H_16_PcZn, and (*t*-Bu)_4_PcZn orientation on gold surfaces. The space-filling models are based on the Pcs single-crystal X-ray structures. The F and the central metal atoms are shown as van der Waals spheres. The rest of the atoms are shown in capped-stick representations. Color code: H, white; C, black; N, blue; F, green. *Top row*. Face-down orientation of the nonpolar molecules exhibiting 4-fold symmetry adsorbed on gold. *Middle and Bottom rows.* Two of the possible edge-on head-to-tail orientations of the polar F_40_PcZn (middle row) and F_52_PcZn (bottom row) adsorbed on gold. Side views are shown in the last two panels of each row. The antiparallel orientation of the molecular dipole vectors, not on scale, is also shown.

For F_64_PcZn on Au, the maximum contact angles of hexadecane were relatively low, 24°/10° (adv/rec), but close to the values we reported for the monolayers of F_64_PcZn supported on mica and on poly­(ethylene terephthalate) substrates.^30^ The common molecular geometry was the “face-down” orientation of F_64_PcZn, exposing a polar, highly acidic Lewis metal center, resulting in lower advancing and, especially, receding contact angles for both water and hexadecane. The surfaces of gold-supported H_16_PcZn and F_16_PcZn, in the absence of oleophobic *R*_f_ groups, were completely wettable by hexadecane, while the presence of oleophobic *t*-C_4_H_9_ groups rendered (*t*-Bu)_4_PcZn surfaces partially wettable by hexadecane (12°/0°, adv/rec).

The orientation of molecules appeared puzzling: why did the molecules with the lowest and highest numbers of bulky *R*_f_ groups, *z* = 0 and 4, respectively, adopt the “face-down” configuration, while those of intermediate bulkiness, *z* = 2 and *z* = 3, adopt the “edge-on” one? To rationalize the observed differences, the symmetry and the polarity of the molecules must be considered. The special behavior of F_40_PcZn and F_52_PcZn, the C_2v_ complexes, was attributed to the absence of an inversion center, present in the D_4h_ complexes, which resulted in the appearance of a permanent dipole moment, Table 1. Conceivably, dipole–dipole interaction along with steric repulsion of the wedge-shaped molecules F_40_PcZn and F_52_PcZn drove the “edge-on-head-to-tail” arrangement in the adsorbed state, with the antiparallel orientation of molecules observed in their bulk solids, Figure S6. In the absence of a dipole moment (*z* = 0, 4), the strong Zn–Au (*z* = 0) or, in the absence of Zn–Au interactions (*z* = 4), weak van der Waals interactions resulted in the “face-down” configuration. For the *z* = 2 and 3, i.e., F_40_PcZn and F_52_PcZn, respectively, the Zn–Au interactions were largely absent, but the intermolecular dipole–dipole interactions appeared as the next strongest force, and thus the determining one in a monolayer film, resulting in the “edge-on” configuration. In the absence of strong molecule–substrate or molecule–molecule interactions (e.g., ionic, covalent, donor–acceptor, H-bonding, etc.), the steric and dipole interactions can play the leading role in the surface assemblies.^55,56^ The importance of dipole–dipole interactions as a major factor determining the orientation of adsorbed molecules and as a driving force for the assembly of surface superstructures has been demonstrated for a wide variety of systems.^57–62^ However, it should be emphasized that linking the presence of fluorinated metal-centered aromatic molecules to the specific architecture of their molecular assemblies is complicated by several factors whose relative importance is not fully understood. For instance, the metal centers of F*_n_* PcM molecules exhibit progressively stronger Lewis acidity as *n* increases, leading to a molecularly amphiphilic, detergent-like character.^30^ While the metal center is increasingly prone to coordinate surface electron-rich atoms (such as oxygen), or directly interact with metallic surfaces, including Au^63^ this tendency is simultaneously diminished by increased molecular steric hindrance for *n* > 16.

Surface coordination of the metal introduces a dipole component of *n*-dependent magnitude perpendicular to the aromatic ring, resulting in a total molecular dipole moment that forms an *n*-dependent angle with the plane of the macrocycle. The complex interplay between metal coordination strength and the magnitude/orientation of the molecular dipoles—all exacerbated by the presence of F and dependent on the value of *n*—renders an*a priori* prediction of the surface orientation of fluorinated metal phthalocyanines difficult. Additionally, X-ray structures reveal F---F intermolecular interactions below the sum of their van der Waals radii 2.94 Å, suggesting nonbonding interactions that favor long-range order and crystallinity. Taken together, these data underscore the difficulty of defining a general, clear-cut structural role for dipole moments.

The energetics of the Pcs adsorption interactions were provided by the adsorption isotherms. For the adsorption on planar Au surfaces, the change in the solution concentration of Pcs was negligible and could not be used to determine the adsorption. Instead, the adsorption isotherms of Pcs were constructed from the XPS thickness data. The amount of the Pcs adsorbed Γ was obtained from the adsorbed thickness (*t*) and the molecular volume of the adsorbed molecules (*V*_m_) as follows

3 $$\Gamma \left[\frac{mol}{m^{2}}\right]=t/{(V}_{m}\cdot N_{A})$$

where *N*_A_–Avogadro’s constant. As no assumptions were made regarding the orientation of the adsorbed molecules, eq ([Disp-formula eq3]) was valid regardless of the adsorbed geometry (e.g., “face-down” vs “edge-on”). The adsorption isotherms, Figure S7, were *L*-type (Langmuir) according to the standard classification.^64^ A gradual increase in adsorption followed by a plateau after the solution concentration reached ∼50–100 μM was observed. For the poorly soluble H_16_PcZn, the adsorption increased roughly linearly up to 40 μM, the highest solution concentration. The adsorption equilibrium constants (*K*_ads_) were obtained from the linear (Henry) portion of the isotherms (See Supporting Information for details). Heats of adsorption (*q*) were determined from the temperature dependence of *K*_ads_ using van’t Hoff plots. The results are summarized in Table 3.

**Table 3:** Thermodynamics of the Pcs Adsorption on Au

	Phthalocyanine					
	H_16_PcZn	(*t*-Bu)_4_PcZn	F_16_PcZn	F_40_PcZn	F_52_PcZn	F_64_PcZn
The Henry adsorption constant^a^ (*K*_ads_), 10^4^	4.0(0.2)	3.7(0.2)	3.9(0.2)	4.8(0.2)	5.2(0.2)	2.2(0.2)
The heat of adsorption^a^ (*q*), kJ/mol	–6.0(0.5)	–4.0(0.5)	–11.3(0.8)	–17(1)	–19(1)	–2.1(0.3)

a Standard errors are given in parentheses.

The values of the adsorption constants, ∼10^4^, indicated spontaneous adsorption in all cases. The heats of adsorption were negative (exothermic) for all Pcs. Notably, the adsorption heats were substantially smaller than the ∼100 kJ/mol values reported for the adsorption of Pcs on metals in vacuum,^31^ which was attributed to the solvation of Pcs and the surface. The largest values of *K*_ads_ and heats of adsorption (by absolute values), observed for noncentrosymmetric F_40_PcZn and F_52_PcZn, were attributed to the sum of dipole–dipole and van der Waals interactions, as per the “edge-on-head-to-tail” model, Figure 8. Image (mirror) dipoles and interface dipoles produced by adsorbed polar molecules on metals could also contribute.^65^ The centrosymmetric Pcs, H_16_PcZn and F_16_PcZn, have intermediate *q* values, due to a combination of van der Waals type and specific Zn–Au interactions, as mentioned above. The heats of adsorption of bulky Pcs (*t*-Bu)_4_PcZn and F_64_PcZn were the lowest in the series (at or below RT level), regardless of the electron-poor or electron-rich nature of the Pc, as Zn–Au interactions were precluded sterically. For these bulky Pcs, the driving force adsorption was thus purely entropic, based on the displacement of a large number of solvent (ethanol) molecules from the interface.

## Conclusions

The efficient deposition of monolayers of a series of metal–organic phthalocyanines exhibiting a systematic variation of electronic and steric properties on gold was achieved using a simple equilibrium solution adsorption method. The variation of experimentally determined XPS thicknesses, adsorption equilibrium constants, heats of adsorption, and surface wettability (contact angles) varied congruently with Pcs calculated intrinsic dipole moments, demonstrating its critical role in the arrangement of the Pcs monolayer structures, Figure 9.

**Figure 9:**
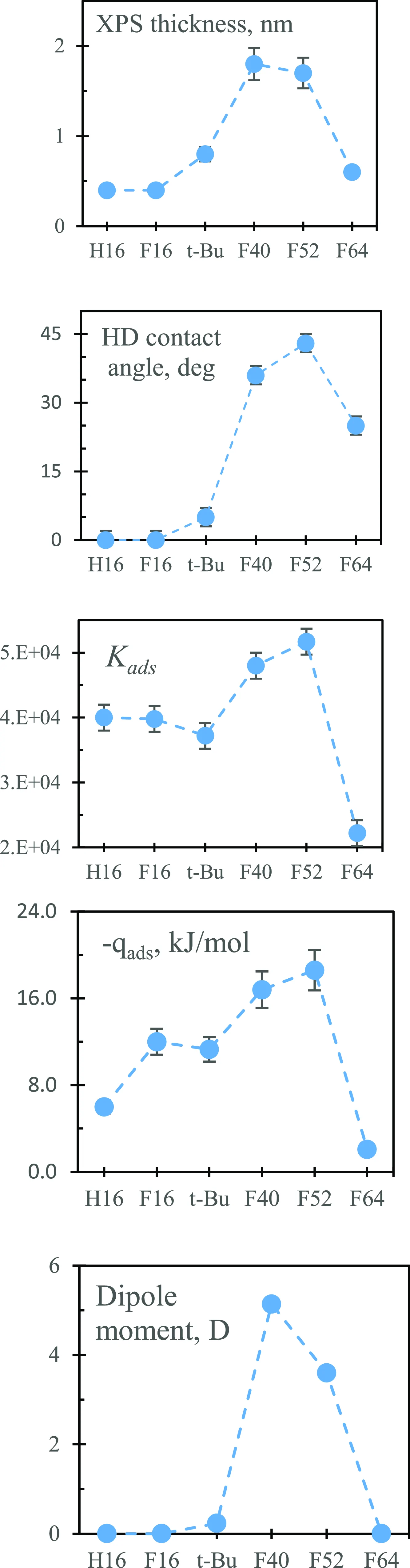
Summary of physical properties of the Pcs adsorbed on Au, plotted along with the calculated dipole moments of pure Pcs.

The experimentally based modeling strategy and the recognition of the importance of dipole–dipole interactions for the surface assembly of these molecules, following the resolution of forces that determine the structure of Pcs thin films, might be used to provide insights for other surface science studies.

## Supplementary Material



## Notes

C 1s peak fit parameters, Zn 2p and N 1s core level spectra, contact angles, methodology of obtaining the adsorption equilibrium constants from the isotherms, and X-ray structures of polar fluorinated Pcs dimers (PDF)
