# Supplementary material for: Symmetry and Type of Peripheral Substituents Tune the Architecture of Gold-Supported Fluoro Phthalocyanine Films
Source: Langmuir. 2026 Sep 21;42(39):28927–38. doi: 10.1021/acs.langmuir.6c02868 (PMC13648273; doi:10.1021/acs.langmuir.6c02868)
Supplement: Supplementary file 1 [file la6c02868_si_001.pdf]

Supplement data to

Symmetry and Type of Peripheral Substituents Tune the Architecture of Gold-supported Fluoro Phthalocyanine Films

Abdul Azeez[1,2], Jonathan Pinnock[1], Usha Kalra[1], Kai Hansen[1], Sanskruti Brahmbhatt[1], Hemantbhai H. Patel[1,2], Marius Pelmuş[1,2]*, Sergiu M. Gorun[1,2]*, and Alexander Y. Fadeev[1]*

[1]Department of Chemistry and Biochemistry, Seton Hall University, South Orange, NJ 07079, USA

[2]Center for Functional Materials, Seton Hall University, South Orange, NJ 07079, USA

Figure S1. Plot of the calculated van der Waals molecular volumes (nm^3/molecule) of all PcZn complexes reported here vs. their molecular volumes obtained from XRD data. Estimated standard deviation values of fitting parameters provided in the fitting linear equation are given in parentheses.

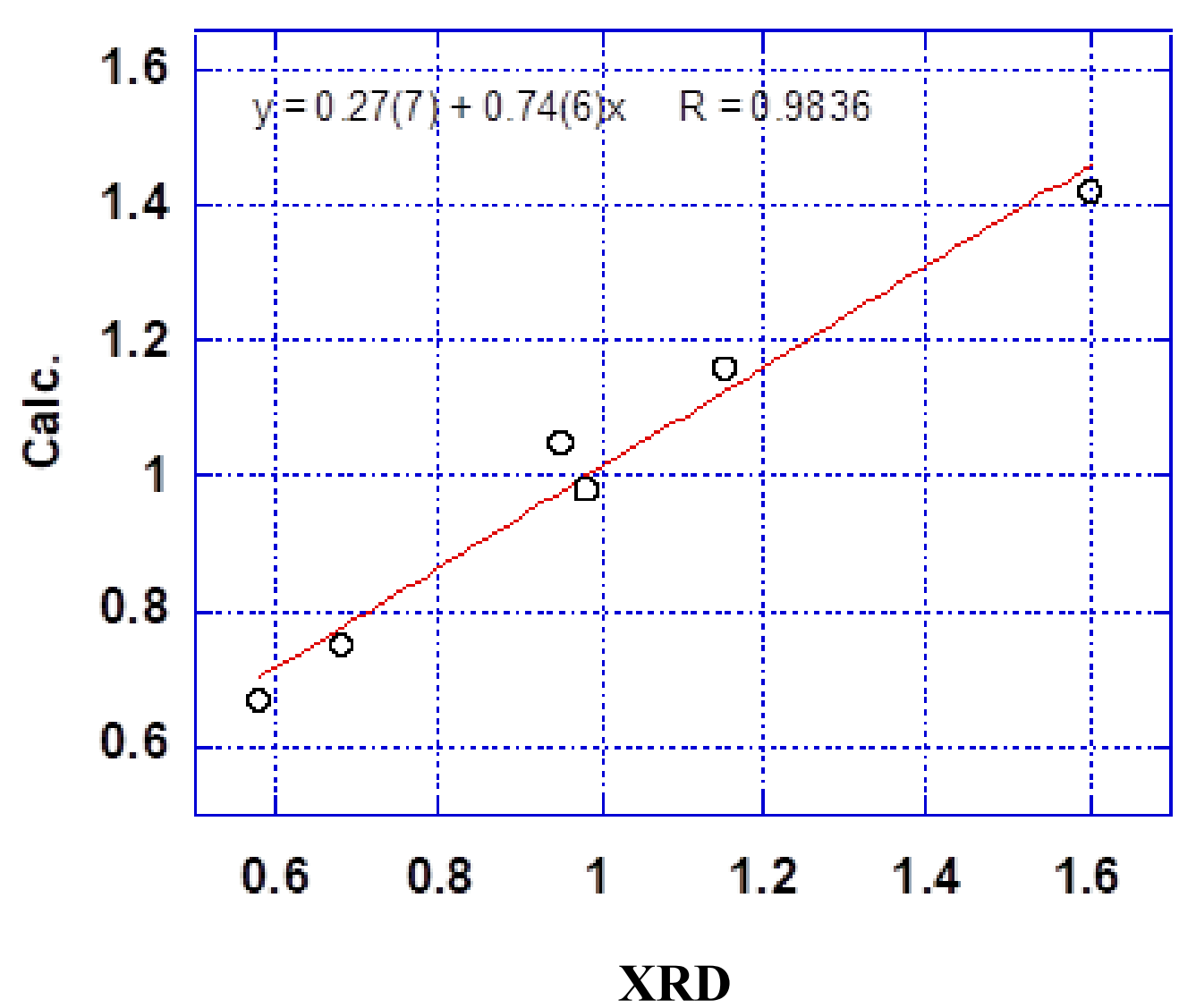

Table S1. Summary of the C1s peak fit analysis for the bulk Pcs.

	Peak Assigned	BE* (eV)	FWHM (eV)	Peak area* (%)	Formula (%)
$F_{64}PcZn$	CC	284.8	1.7	25	28
	S_{CC}	286.7	1.5	3	
	CN	286.3	1.3	12	14
	S_{CN}	288.6	1.4	2	
	CF_{sp2}	287.2	1.2	12	14
	S_{CFsp2}	290.6	1.4	2	
	CF_{sp3}	288.6	1.5	12	14
	S_{CFsp3}	291.6	1.5	2	
	CF_3	293.5	1.5	25	28
	S_{CF3}	295.0	1.5	3	
$F_{52}PcZn$	CC	284.6	1.6	26	28
	S_{CC}	285.4	1.4	2	
	CN	286.1	1.3	19	20
	S_{CN}	287.9	1.3	1	
	CF_{sp2}	287.4	1.3	15	16
	S_{CFsp2}	298.8	1.3	1	
	CF_{sp3}	288.9	1.6	12	12
	S_{CFsp3}	291.3	1.4	1	
	CF_3	293.0	1.3	21	24
	S_{CF3}	295.0	1.3	2	
$F_{40}PcZn$	CC	284.6	1.6	27	27
	S_{CC}	285.4	1.5	1	
	CN	286.3	1.2	18	18
	S_{CN}	287.9	1.4	1	
	CF_{sp2}	287.5	1.1	27	27
	S_{CFsp2}	289.9	1.2	1	
	CF_{sp3}	289.1	0.9	9	9
	S_{CFsp3}	290.9	1.1	1	
	CF_3	292.8	1.2	14	18
	S_{CF3}	294.8	1.1	1	
$F_{16}PcZn$	CC	284.3	1.1	22	25
	S_{CC}	286.0	1.3	3	
	CN	285.2	2.1	22	25
	S_{CN}	286.9	1.4	3	

	CF_{sp2}	287.2	2.3	45	50
	S_{CFsp2}	288.7	1.6	4	
$H_{16}PcZn$	CC	284.6	1.7	73	75
	S_{CC}	286.4	1.8	1	
	CN	286.2	2.0	23	25
	S_{CN}	288.1	2.1	3	
$(t\text{-}Bu)_4PcZn$	CC_{sp2}	284.5	1.1	45	50
	S_{CCsp2}	286.3	1.1	4	
	CC_{sp3}	285.2	1.0	31	33
	S_{CCsp3}	287.0	1.1	3	
	CN	285.7	1.0	16	17
	S_{CN}	286.5	1.1	2	

*Average numbers are reported for duplicate measurements, the error in the peaks positions was estimated to be 0.05 eV, while the relative error in the peaks areas was 5-7%.

Figure S2. Zn2p core level spectra for bulk Pcs (solid lines) vs Pcs adsorbed on gold (dashed lines). The Pcs are labeled as per their abbreviated substituents designations.

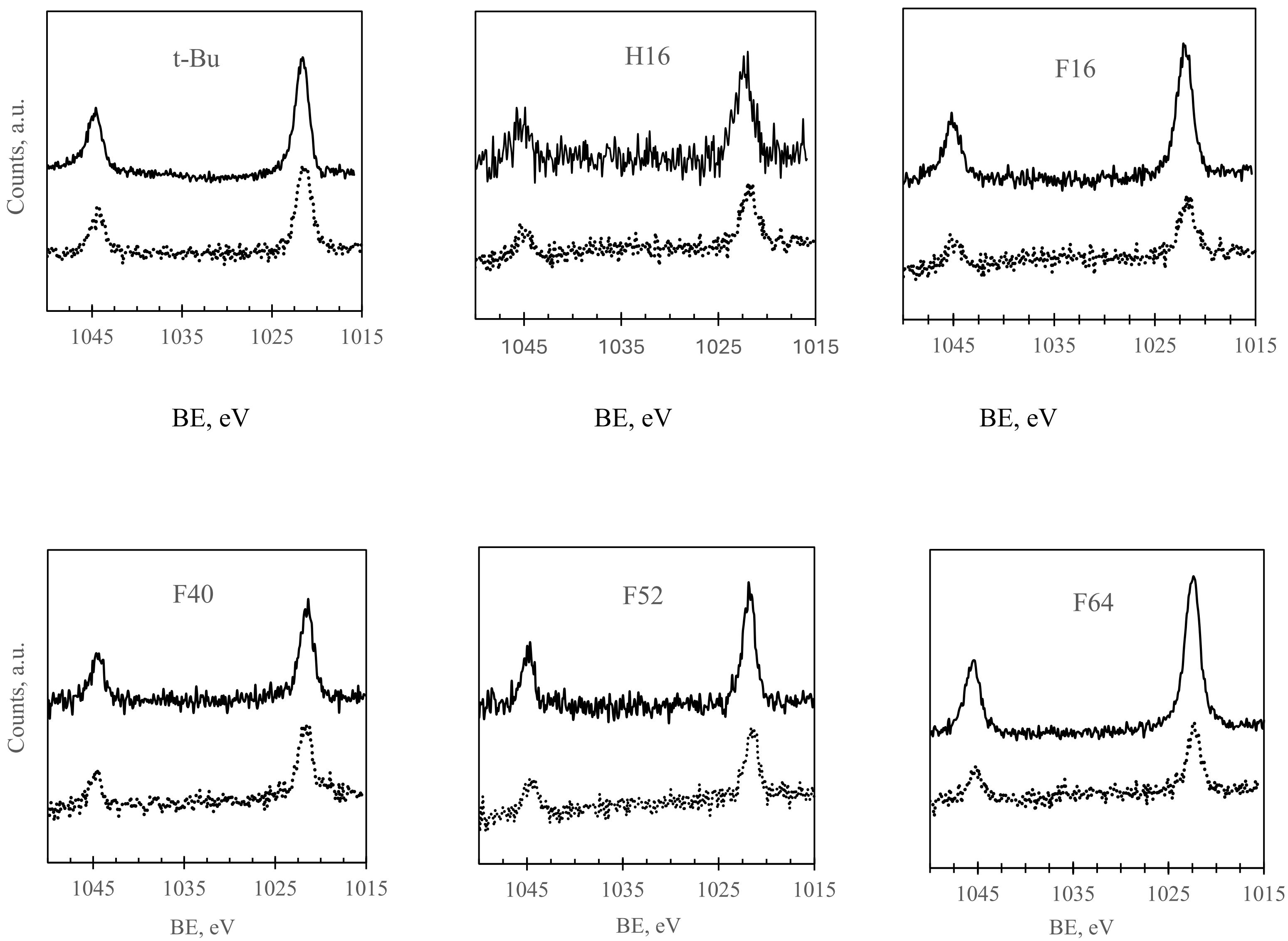

Figure S3. N1s core level spectra for bulk Pcs (solid lines) vs Pcs adsorbed on gold (dashed lines). The Pcs are labeled as per their abbreviated substituents designations.

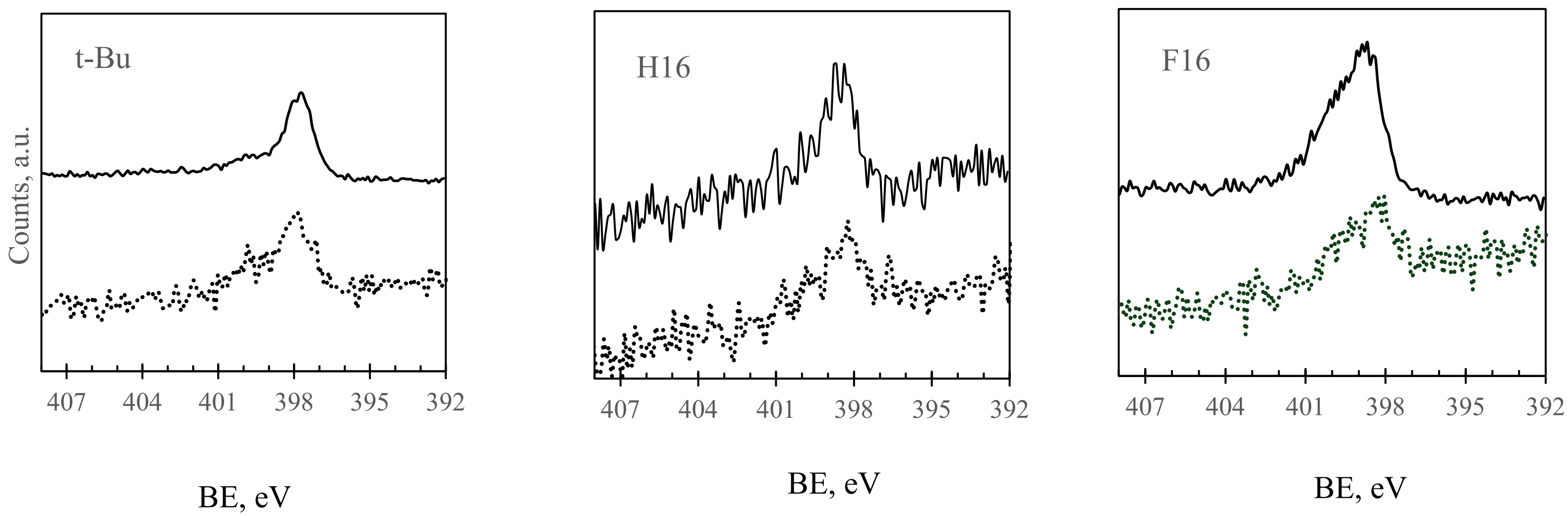

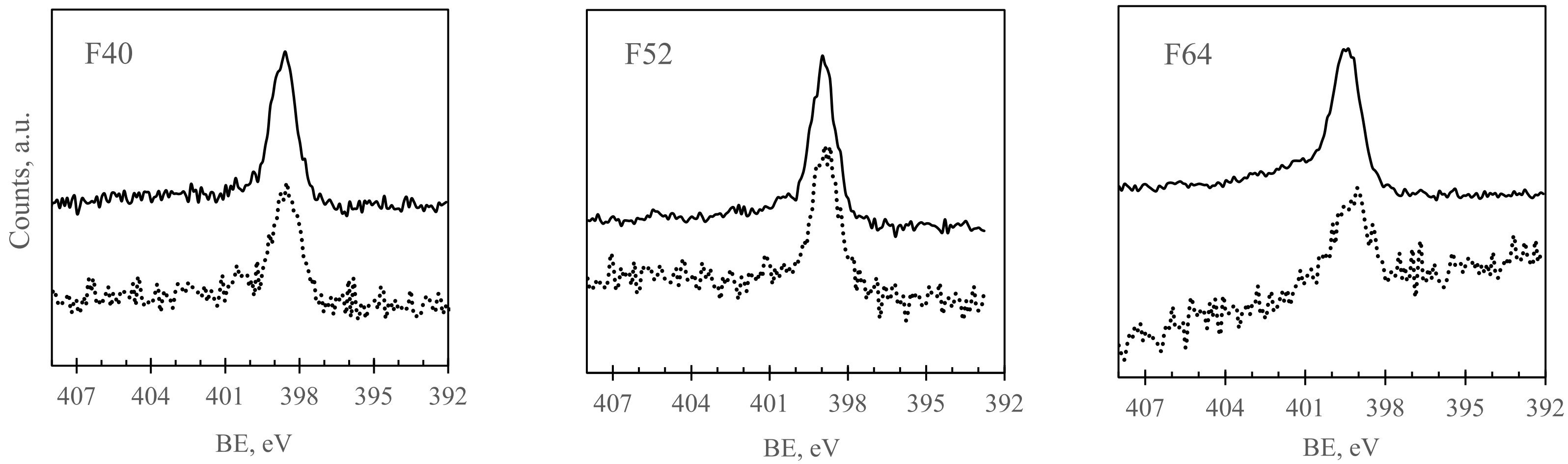
F40
F52
F64
Counts, a.u.
407
404
401
398
395
392
BE, eV

Figure S4. Plot of N1s binding energy vs. the number n of F atoms in F_nPcZn. The increase can be fit with a 2nd degree polynomial.

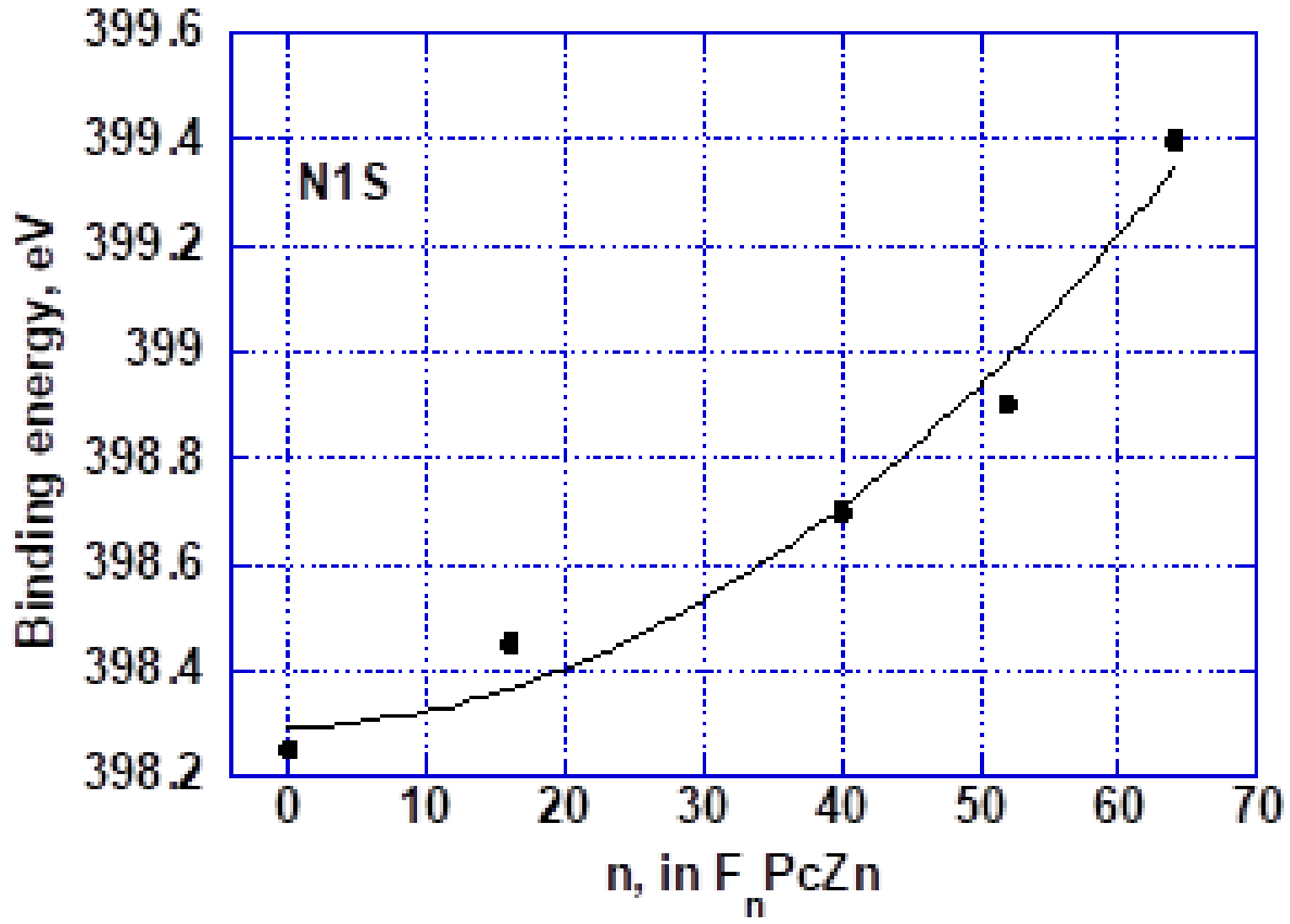

Figure S5. Water (solid lines) and hexadecane (dashed lines) advancing contact angles of gold surfaces exposed to different concentrations of Pcs in ethanol. The Pcs are labeled as per their abbreviated substituents designations.

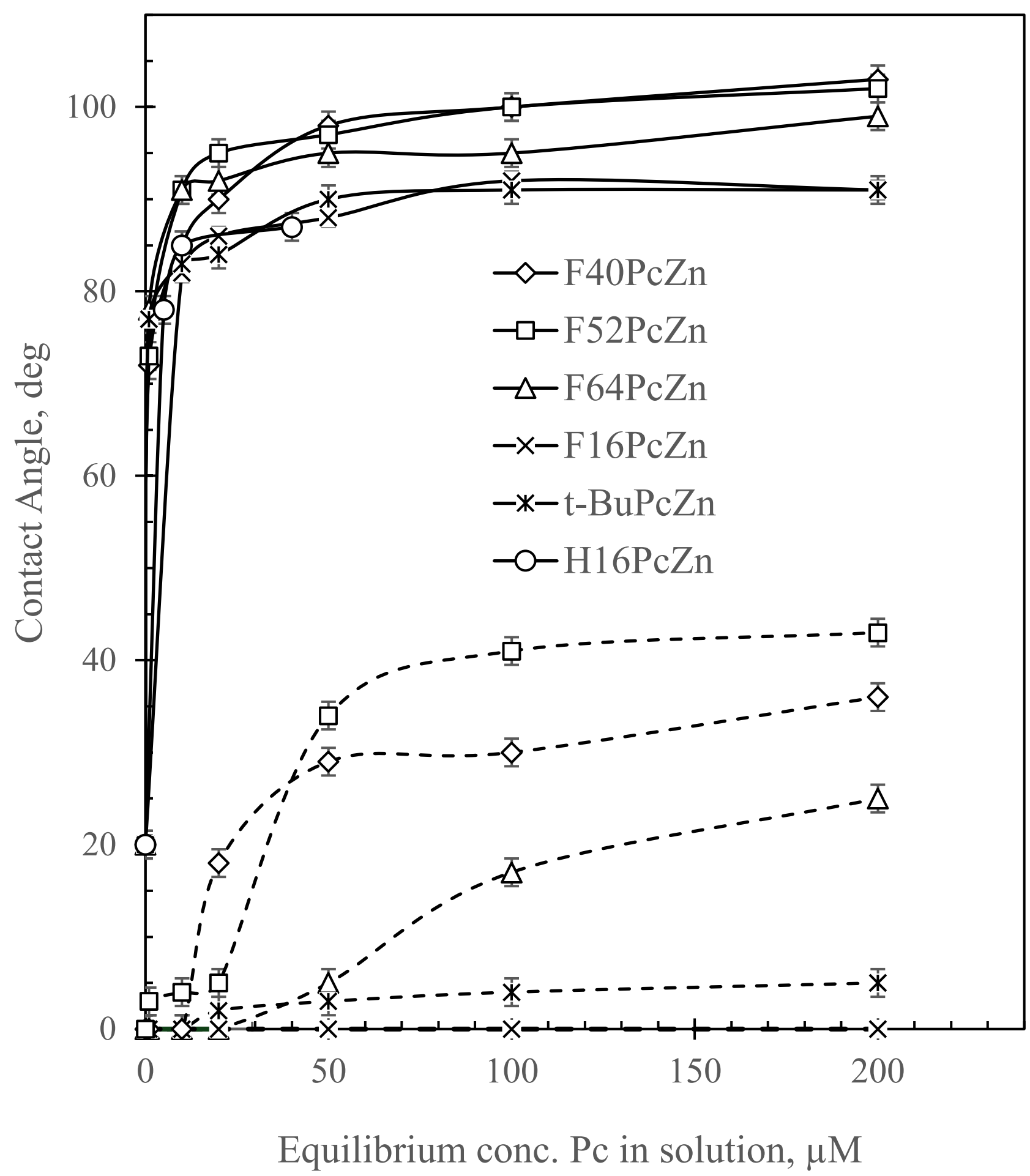

Figure S6. Depictions of F_{40}PcZn and F_{52}PcZn interactions in the crystalline state. Axial ligands and solvents of crystallization have been omitted. Graphical representation types and color code: Zn, ball-and-stick, orange; F, capped sticks, green; N, capped sticks, blue; C, capped sticks, black. a) The structure of F_{40}PcZn showing the direction of the dipole moment. The dipole vectors magnitudes are not on scale. b) Side view of the F_{40}PcZn dimer formed by the overlap of the two quadrants that do not bear R_f groups. c) van der Waals (space filling) representation of the F_{40}PcZn dimer. d) Overlap of dimers and direction of the dipole vectors. e) The structure of F_{52}PcZn showing the direction of the dipole moment. The dipole vectors magnitudes are not on scale. f) Side view of the F_{52}PcZn dimer formed by the overlap of the Pc quadrants that do not bear R_f groups. g) van der Waals (space filling) representation of the F_{52}PcZn dimer. h) Overlap of dimers and the direction of the dipole vectors. In projection, shown here, the antiparallel dipole vectors appear colinear, but they are not, as they belong to different molecules.

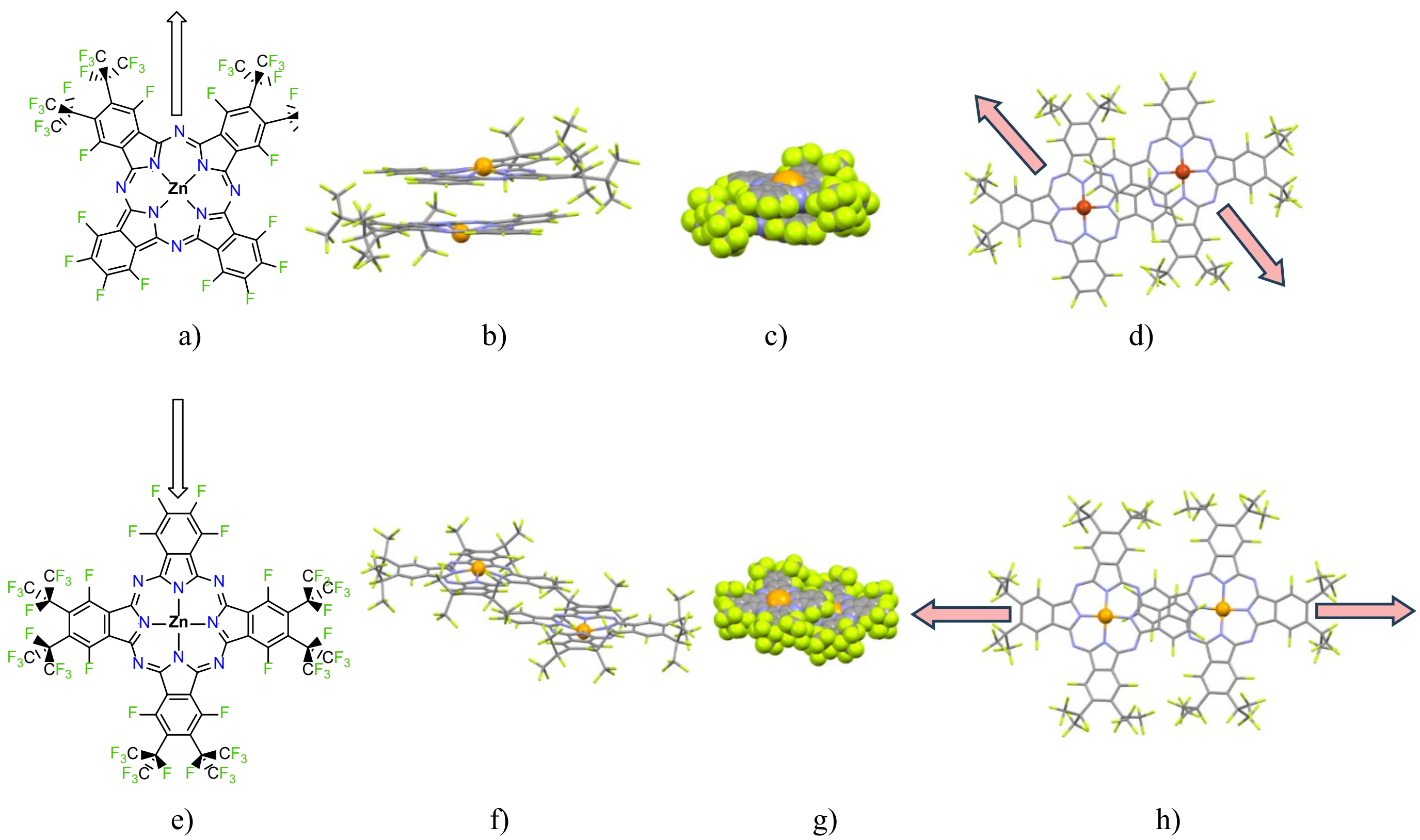

Determination of the adsorption equilibrium constants.

Following Everett[1], the adsorption equilibrium for a two-component system (component 1 – adsorbate and component 2 - solvent) can be written as follows:

$$(1)_l + (2)_s \leftrightarrow (1)_s + (2)_l$$

where subscripts *l* and *s* refer to liquid and surface states, respectively. The expression for the equilibrium constant is given as:

$$K = \frac{x_{1,s} \cdot x_{2,l}}{x_{1,l} \cdot x_{2,s}} \qquad (1)$$

where x_i are molar fractions of the components: x_1 – molar fraction of phthalocyanine, x_2 – molar fraction of solvent (ethanol). For adsorptions from dilute solutions (μM range), and at low surface coverage (initial portion of the isotherm), the molar fractions of solvent in the solution ($x_{2,l}$) and on the surface ($x_{2,s}$) are very close to 1. Thus, eq. 1 simplifies to:

$$K \approx \frac{x_{1,s}}{x_{1,l}} \qquad (2)$$

Using parameters determined experimentally, eq. 2 can be written:

$$K = \frac{\Gamma_1 \cdot \sigma_2}{C_1 \cdot v_2} \qquad (3)$$

Where Γ_1 is the adsorption of phthalocyanines at the equilibrium solution concentration C_1, σ_2 – molecular cross-section and v_2 – molecular volume of the solvent. Using the molecular dimensions of ethanol obtained from its liquid density, σ_2 = 0.23 nm^2, v_2 = 0.097 nm^3, eq.3 simplifies to:

$$K = 2.371 \cdot \frac{\Gamma_1}{C_1} \qquad (4)$$

In eq. 4, Γ_1 is expressed in μmole/m^2, C_1 – mol/L, and coefficient 2.371 has units nm^{-1}. The equilibrium constant determined by eq. 4 is dimensionless. This approach eliminates the problem of "dimensional inconsistency", which is common in the literature, for a review see Ref. 2.

The heat of adsorption (q) was determined from the temperature dependence of the adsorption equilibrium constant by the van't Hoff equation:

$$lnK = -\frac{q}{RT} + const \qquad (5)$$

To use the eq. 5, the adsorption isotherms were measured at three different temperatures: 253, 298 and 333 K, and q was obtained as the slope of the plot *lnK* vs *1/T*. All the adsorption measurements were conducted in two independent series for statistical analysis. The errors for the equilibrium constants were in the 5-10% range. The errors for the heats of adsorption were in the 5-15% range.

Figure S7. Adsorption isotherms of Pcs on gold. The Pcs are labeled as per their abbreviated substituents designations.

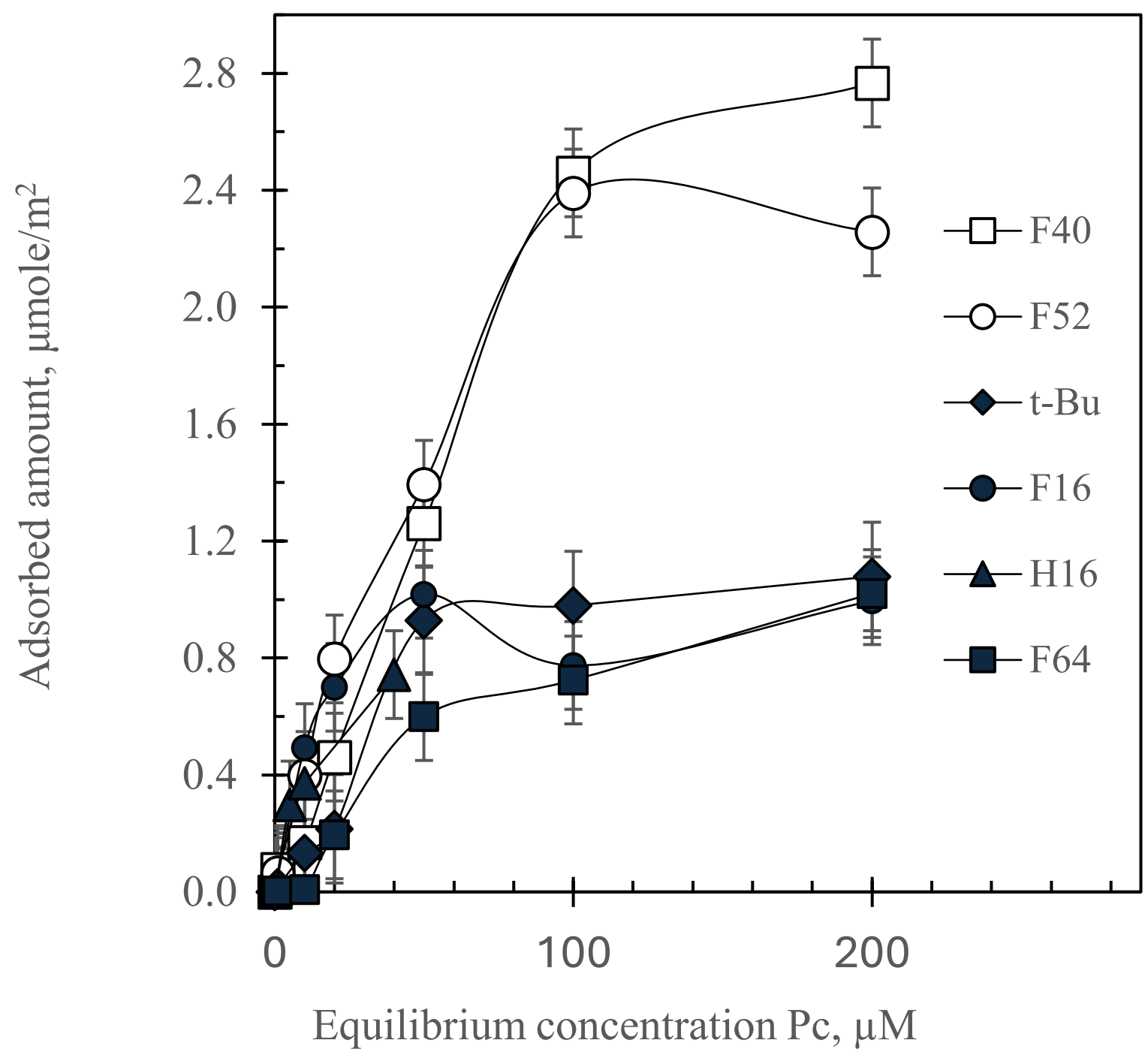

References

1. Everett, D.H. Thermodynamics of adsorption from solution. Part 1. Perfect systems. *Transactions Faraday Society* **1964**, *60*, 1803-1813. DOI.org/10.1039/tf9646001803.
2. Lima, E.C., Hosseiniso-Bandegharaei, A., Moreno-Piraján, J.C., Anastopoulos, I. A critical review of the estimation of the thermodynamic parameters on adsorption equilibria. Wrong use of equilibrium constant in the Van't Hoof equation for calculation of thermodynamic parameters of adsorption. *Journal of Molecular Liquids* **2019**, *273*, 425–434. DOI.org/10.1016/j.molliq.2018.10.048.
